# Genetically programmable cell membrane-camouflaged nanoparticles for targeted combination therapy of colorectal cancer

**DOI:** 10.1038/s41392-024-01859-4

**Published:** 2024-06-12

**Authors:** Yun Yang, Qingya Liu, Meng Wang, Lang Li, Yan Yu, Meng Pan, Danrong Hu, Bingyang Chu, Ying Qu, Zhiyong Qian

**Affiliations:** Department of Biotherapy, Cancer Center and State Key Laboratory of Biotherapy, West China Hospital, Sichuan University, Chengdu, 610041 China

**Keywords:** Drug development, Drug delivery

## Abstract

Cell membrane-camouflaged nanoparticles possess inherent advantages derived from their membrane structure and surface antigens, including prolonged circulation in the bloodstream, specific cell recognition and targeting capabilities, and potential for immunotherapy. Herein, we introduce a cell membrane biomimetic nanodrug platform termed MPB-3BP@CM NPs. Comprising microporous Prussian blue nanoparticles (MPB NPs) serving as both a photothermal sensitizer and carrier for 3-bromopyruvate (3BP), these nanoparticles are cloaked in a genetically programmable cell membrane displaying variants of signal regulatory protein α (SIRPα) with enhanced affinity to CD47. As a result, MPB-3BP@CM NPs inherit the characteristics of the original cell membrane, exhibiting an extended circulation time in the bloodstream and effectively targeting CD47 on the cytomembrane of colorectal cancer (CRC) cells. Notably, blocking CD47 with MPB-3BP@CM NPs enhances the phagocytosis of CRC cells by macrophages. Additionally, 3BP, an inhibitor of hexokinase II (HK_2_), suppresses glycolysis, leading to a reduction in adenosine triphosphate (ATP) levels and lactate production. Besides, it promotes the polarization of tumor-associated macrophages (TAMs) towards an anti-tumor M1 phenotype. Furthermore, integration with MPB NPs-mediated photothermal therapy (PTT) enhances the therapeutic efficacy against tumors. These advantages make MPB-3BP@CM NPs an attractive platform for the future development of innovative therapeutic approaches for CRC. Concurrently, it introduces a universal approach for engineering disease-tailored cell membranes for tumor therapy.

## Introduction

CRC ranks third among global malignancies, with the second highest mortality rate attributable to cancer in 2020.^1^ Due to its high morbidity and mortality, it is estimated that new CRC cases will reach over 2.2 million with 1.1 million fatalities globally by 2030.^2^ Meanwhile, because early-stage CRC is often asymptomatic, patients with CRC are usually diagnosed at intermediate or advanced stages, thus missing the opportunity for curative treatment.^1^ At present, surgical intervention combined with chemotherapy and radiotherapy is a priority treatment strategy for intermediate and advanced CRC. While traditional post-surgery chemotherapy and radiotherapy have shown some effectiveness in enhancing therapeutic outcomes, they are frequently marred by adverse reactions and instances of treatment ineffectiveness.^3^ Therefore, there is an urgent demand to devise safer and more effective approaches for treating CRC.

Recently, metabolic reprogramming has emerged as a distinctive feature of cancer, characterized by increased activation of glycolysis and heightened lactate fermentation.^4,5^ Cancer cells undergo significant metabolic alterations to facilitate efficient nutrient utilization and biomass generation, sustaining their rapid proliferation and other malignant characteristics.^6^ Therefore, disruption of glycolysis is regarded as a promising therapeutic approach in CRC.^7,8^ As a glycolytic inhibitor, 3BP demonstrates potent inhibition of HK_2_, an enzyme responsible for the ATP-dependent initiation of glycolysis. Overexpression of HK_2_ is observed in numerous cancer types, contributing to enhanced glycolytic activity.^9,10^ The efficacy of 3BP in anticancer therapy has been demonstrated through animal models, exhibiting no apparent side effects.^11^ Nevertheless, due to its alkylating properties, free 3BP is unstable and controversial when delivered systemically, which has impeded its clinical development and use as a drug for cancer treatment.^12^ Thus, there is a clear need to develop a new 3BP formulation with systemic delivery capability. Meanwhile, it is becoming evident that distinct metabolic preferences and dependencies are present among various tumor types, and even within tumors originating from the same tissue.^13,14^ Consequently, the therapeutic effectiveness of glycolysis inhibitors has been constrained, prompting exploration into combination therapies that hold promise in cancer treatment.^15–17^

PTT is widely considered for medical applications because of its unique advantages such as minimally invasive process, promising tumor ablation, and few side effects.^18^ Prussian blue nanoparticles, renowned for their favorable biocompatibility and distinctive characteristics, have been extensively investigated as drug delivery carriers, photothermal agents, and contrast agents in cancer therapy.^19,20^ MPB NPs contain large cavities within a single crystal,^21^ making them a promising delivery carrier for 3BP with improved stability and therapeutic efficacy in vivo. Unfortunately, unmodified MPB NPs are prone to clearance by the immune system and premature drug leakage before reaching target sites, leading to compromised therapeutic efficacy and heightened toxic side effects.^22,23^ Enhancing immune evasion and disease-specific targeting has garnered growing attention, resulting in a surge of research on cell membrane-camouflaged nanoparticles. These innovative nanoparticles consist of a synthetic nanoparticle core enveloped by a natural cell membrane layer. By mimicking the characteristics of their cellular source, cell membrane-camouflaged nanoparticles inherently possess diverse capabilities, such as prolonged circulation times, precise cancer cell identification and targeting, and potential applications in immunotherapy.^24^ However, obtaining these natural cell membranes in large quantities for clinical applications is difficult, and they may have limited functions. Therefore, to overcome these limitations, developing individually customized cell membranes with functionalized membrane proteins using genetic editing has become a promising area.^25–27^ CD47, a ‘marker-of-self’ protein that is highly overexpression on the cytomembrane of CRC cells compared to normal tissues, has been shown to protect CRC cells from macrophage phagocytosis by transmitting a ‘don’t eat me’ signal through the SIRPα receptor.^28,29^ Accordingly, blocking the SIRPα-CD47 interaction can enhance the phagocytic ability of macrophages toward cancer cells, while CD47 also serves as binding sites for nanoparticles targeting CRC cells. Nonetheless, the wild-type SIRPα has a weak affinity for CD47. To overcome this limitation, high-affinity human SIRPα monomers (MSIRPα) were engineered, exhibiting approximately a 50,000-fold higher affinity for CD47 compared to wild-type SIRPα. Moreover, MSIRPα effectively antagonized CD47 on cancer cells without inducing toxicity in normal cells expressing CD47.^30^

**Scheme 1:**
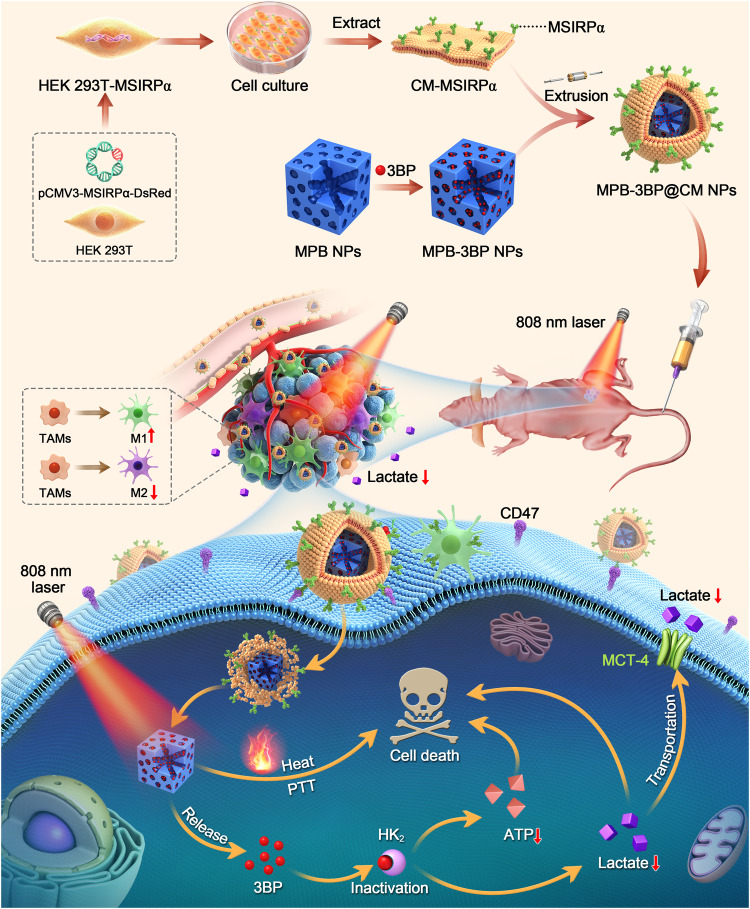
The construction of MPB-3BP@CM NPs and their implementation in combined therapy for CRC

Inspired by these findings, we developed a hybrid nanoparticle that utilizes MPB NPs to encapsulate 3BP in their cavities, and coated it with genetically programmable cell membrane overexpressing MSIRPα (CM-MSIRPα) to synchronously recognize and eradicate tumors (Scheme 1). The resulting novel nanoparticles, henceforth designated as MPB-3BP@CM NPs, exhibited significantly enhanced antitumor activity for several reasons. First, MPB-3BP@CM NPs with CM-MSIRPα cloaking displayed long blood circulation ability and efficient tumor-site targeting, concurrently mitigating systemic infusion-induced adverse effects. Meanwhile, the presence of more enriched MPB-3BP@CM NPs in tumor tissue competitively blocked the SIRPα-CD47 interaction between tumor cells and macrophages, thus accentuating macrophage phagocytosis of cancer cells. Second, 3BP disrupted the energy supply by inhibiting glycolysis, potentially leading to starvation-induced apoptosis. It also simultaneously reduced tumor lactate production and polarized TAMs toward an antitumor M1 phenotype,^31–34^ which combined with the blockade of the SIRPα-CD47 interaction, accentuated macrophage phagocytosis of cancer cells. Third, the accumulation of MPB-3BP@CM NPs at the tumor tissue may have permitted the use of photoacoustic imaging (PAI) for diagnosis, which provided an optimal timing for in vivo PTT and ensuring highly effective tumor ablation. Ultimately, the findings from both in vitro and in vivo experiments underscored the efficacy of MPB-3BP@CM NPs in inhibiting and ablating tumors through a synergistic approach encompassing chemo-photothermal immunotherapy, all while exhibiting minimal systemic toxicity. Hence, the introduction of MPB-3BP@CM NPs offers novel perspectives for advancing the efficacy and safety of CRC treatment strategies.

## Results

### Preparation and characterization of MPB-3BP@CM NPs

**Fig. 1:**
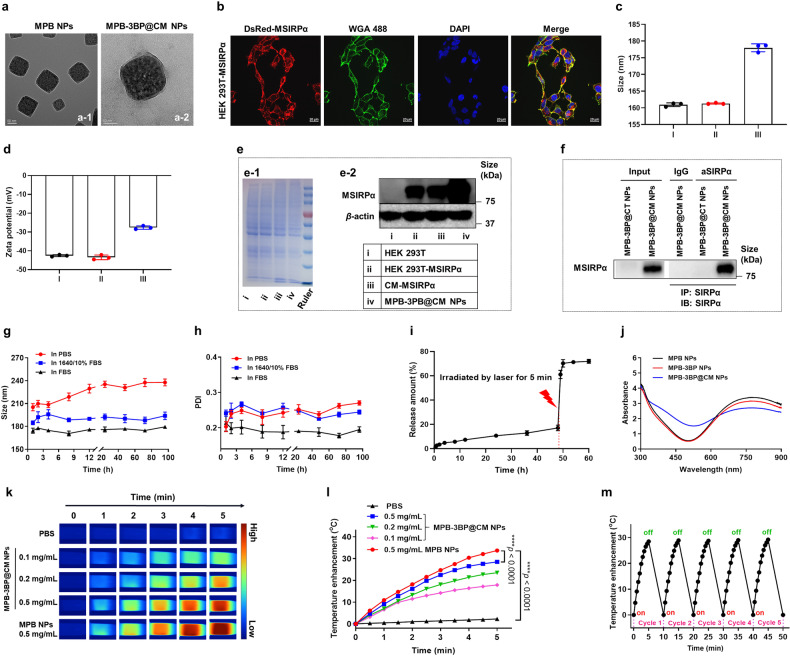
Characterization of MPB-3BP@CM NPs. The TEM images of MPB NPs (**a**-1) and MPB-3BP@CM NPs (**a**-2), scale bar = 50 nm. **b** Establishment of HEK 293T cell stably expressing human MSIRPα receptor on cell membranes (HEK 293T-MSIRPα). WGA 488 dye was utilized to label cell membrane (green channel), DAPI was used to detect the cell nucleus (blue channel). The red channel fluorescence emission originated from DsRed moieties. Scale bar = 20 µm. DLS measured sizes (**c**) and zeta potentials (**d**) of nanoparticles (I: MPB NPs, II: MPB-3BP NPs, III:MPB-3BP@CM NPs). **e**-1 SDS-PAGE protein analysis of HEK 293T cell lysate, HEK 293T-MSIRPα cell lysate, CM-MSIRPα, and MPB-3BP@CM NPs, samples were run at equal protein concentration and stained with coomassie blue. **e**-2 Western blot assay exhibited the expression of human MSIRPα receptor on the HEK 293T cell lysate, HEK 293T-MSIRPα cell lysate, CM-MSIRPα, and MPB-3BP@CM NPs. **f** MPB-3BP@CM NPs were incubated with SIRPα primary antibody overnight and then subjected to immunoprecipitation and western blot assay. DLS measured size (**g**) and PDI (**h**) changes of MPB-3BP@CM NPs incubated in different conditions. **i** In vitro release profiles of 3BP from MPB-3BP@CM NPs. **j** UV−visible spectrum of nanoparticles in deionized water. Infrared thermal images (**k**) and photothermal heating curves (**l**) of MPB-3BP@CM NPs solution under 808 nm laser irradiation at the power density of 1.0 W/cm^2^ for 5 min. **m** Temperature variations of MPB-3BP@CM NPs solution under irradiation with an 808 nm laser at the power density of 1.0 W/cm^2^ for five cycles. All data were presented as mean ± SD (*n* = 3)

The first step in the preparation of the MPB-3BP@CM NPs involved the synthesis of MPB NPs according to a previously published method.^21^ The transmission electron microscopy (TEM) analysis revealed the uniform cubic structure of the synthesized MPB NPs (Fig. 1a-1). Dynamic light scattering (DLS) examination indicated an average hydrodynamic diameter of ~160.9 nm (Fig. 1c) for the MPB NPs, with a polydispersity index (PDI) of around 0.21. Additionally, the zeta potential of the MPB NPs was measured at −42.6 mV (Fig. 1d). Small pores were distinctly observable among the aggregated prussian blue nanocrystals (Supplementary Fig. 1a). The density function theory (DFT) analysis reveals that the pore structure of MPB NPs is comprised of numerous micropores (~1.4 nm) and a few mesopores (~9.2 and ~21.6 nm) (Supplementary Fig. 1b). Additionally, based on the N_2_ absorption-desorption isotherms, the Langmuir surface area of MPB NPs was determined to be 191.0870 m²/g, while the t-Plot micropore volume of pores was measured at 0.049649 cm³/g (Supplementary Fig. 1c). These characterization results evidently confirmed the porous feature of MPB NPs nanostructures prepared, which is crucial for the efficient drug loading. 3BP, which is known as “metabolic catastrophe”, exerts inhibitory effects on glycolysis and demonstrates remarkable efficacy in inducing apoptosis of tumor cells, as well as in preventing tumor growth in preclinical studies.^35^ Subsequently, 3BP was loaded into the MPB NPs cavity to obtain MPB-3BP NPs. After optimization, 1.0 mg of MPB NPs can load 0.176 ± 0.036 mg of 3BP, with an efficiency of 16.8 ± 4.6% (Supplementary Table 1). To prepare CM-MSIRPα, DsRed proteins were fused with MSIRPα receptors (DsRed-MSIRPα) to provide red fluorescence (Supplementary Fig. 2). We subsequently established stable human embryonic kidney 293 T (HEK 293 T) cell lines expressing the MSIRPα receptor on their cell membranes (HEK 293T-MSIRPα). The localization of the MSIRPα receptors was confirmed by labeling the cell membranes with Alexa-Fluor 488 conjugated wheat germ agglutinin (WGA 488). As expected, the red fluorescence of DsRed protein co-localized with green fluorescence of WGA 488 dye on the cellular membranes (Fig. 1b). Furthermore, the HEK 293T-MSIRPα cell line maintained the expression of MSIRPα receptors for over 20 passages (Supplementary Fig. 3). To extract purified CM-MSIRPα, HEK 293T-MSIRPα cells were cultured and subjected to a purification process involving hypotonic lysis, mechanical disruption, and gradient centrifugation to remove intracellular content. The relative content of MSIRPα protein in CM-MSIRPα was 5.2% of the total protein of the cell membrane detected by western blot analysis. To coat the CM-MSIRPα onto the surface of MPB-3BP NPs, the two components were coextruded through 400 and 200 nm polycarbonate porous membranes to obtain MPB-3BP@CM NPs. Using a similar procedure, MPB-3BP NPs coated with cell membrane derived from HEK 293 T cells (MPB-3BP@CT NPs) were prepared. Successively, HEK 293 T cell membrane nanoparticles (CT NPs), CM-MSIRPα nanoparticles (CM NPs), CM-MSIRPα camouflaged MPB NPs (MPB@CM NPs), 3BP-loaded CM-MSIRPα nanoparticles (3BP@CM NPs) were prepared.

**Fig. 2:**
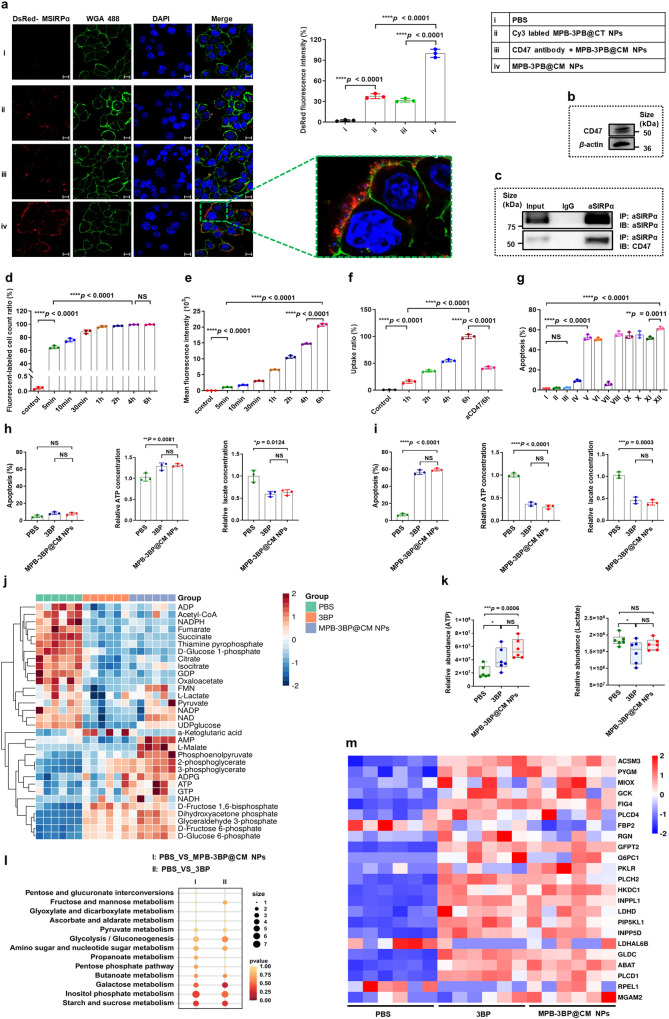
**a** CLSM was employed to visualize the binding of MPB-3BP@CM NPs on the cell membrane of HCT116 cancer cells, while quantification of DsRed-MSIRPα fluorescence intensity (%) was performed based on CLSM observations. WGA 488 dye was utilized to detect the HCT116 cell membrane (green channel) and DAPI was used to detect the cell nucleus (blue channel). The red channel fluorescence emission originated from DsRed moieties. The right image is the enlarged one with the green collar on the left image. Scar bar = 10 µm. **b** Western blot assay exhibited the expression of human CD47 receptors in the HCT116 cell lysate. **c** CO-IP and western blot were employed to examine the interaction between MSIRPα (on MPB-3BP@CM NPs) and CD47 (on HCT116 cells), immunoblot (IB). **d**, **e** Flow cytometric analysis recorded for HCT116 cells incubation with MPB-Cy7.5@CM NPs with varying time intervals, as determined by Cy7.5 channel. **f** The cellular uptake rate was quantified through intracellular Cy7.5 fluorescence pattern observed via CLSM. **g** Quantitative flow cytometry analysis of HCT116 cells apoptosis after different treatments (I: PBS, II: CT NPs, III: CM NPs, IV: MPB NPs + laser, V: 3BP, VI: 3BP@CM NPs, VII: MPB@CM NPs + laser, VIII: MPB-3BP NPs + laser, IX: MPB NPs/3BP/CM NPs + laser, X: MPB-3BP@CT NPs + laser, XI: MPB-3BP@CM NPs, XII: MPB-3BP@CM NPs + laser). The evaluation of apoptosis in HCT116 cells was conducted at 8 h (**h**) and 14 h (**i**) after various treatments, along with the measurement of intracellular ATP and lactate content. **j** Heat map illustrates the heterogeneity in metabolite levels across different groups. **k** The relative levels of ATP and lactate in different groups were quantified by LC-MS. **l** Bubble map were employed to compare the differentially expressed genes associated with metabolism between the control and experimental groups. **m** Heat map illustrates the typical differentially expressed genes associated with metabolism across different groups. All data were presented as mean ± SD (*n* ≥ 3)

The morphology of the MPB-3BP@CM NPs prepared was characterized using TEM. The final MPB-3BP@CM NPs were spherical and displayed a distinctive core-shell structure (Fig. 1a-2 and Supplementary Fig. 4). DLS analysis revealed that the average diameter of the MPB-3BP@CM NPs was around 178.0 nm (Fig. 1c). Coating with CM-MSIRPα changed the zeta potential of MPB-3BP NPs from −43.5 mv to −27.7 mV in MPB-3BP@CM NPs (Fig. 1d). The results of gel electrophoresis followed by protein staining demonstrated that nearly all protein bands of MPB-3BP@CM NPs were preserved compared with CM-MSIRPα and HEK 293T-MSIRPα, confirming the successful functionalization of the nanoparticles with CM-MSIRPα antigens (Fig. 1e-1). Moreover, western blot analysis further indicated that MPB-3BP@CM NPs displayed the MSIRPα receptors (Fig. 1e-2). Besides, MSIRPα receptors were demonstrated to maintain an outside-out orientation on MPB-3BP@CM NPs surfaces using immunoprecipitation (IP) assay (Fig. 1f). In addition, MPB-3BP@CM NPs showed good stability in a physiological environment (Fig. 1g, h).

### In vitro release kinetics

Considering the inherent instability of the aqueous 3BP solution at 37 °C, controlled conditions at 4 °C were selected to evaluate the in vitro release profiles of 3BP from MPB-3BP@CM NPs. The release kinetics of 3BP from MPB-3BP@CM NPs at 4 °C exhibited a slow profile, with only a modest 17.1% cumulative release observed over the course of 48 h (Fig. 1i). Subsequently, the MPB-3BP@CM NPs underwent irradiation with an 808 nm laser at a power density of 1.0 W/cm^2^ for a duration of 5 min. Following a 2-h interval, it was observed that ~70.3% of the encapsulated 3BP was released, indicating that laser irradiation can trigger drug release.

### Photothermal effect and photothermal stability

To investigate the photothermal properties of MPB-3BP@CM NPs, we first investigated their optical absorption properties. The results showed that the nanoparticles retained near infrared (NIR) absorption (Fig. 1j), supporting their use as photothermal agent and photoacoustic contrast agent for cancer therapy. Next, the MPB-3BP@CM NPs solution of varying concentrations was subjected to irradiation using a NIR laser, while monitoring the temperature changes through an infrared thermal camera (Fig. 1k). Analysis of the temperature curves (Fig. 1l) revealed that the temperature of the MPB-3BP@CM NPs solution with a concentration of 0.5 mg/mL increased by 28.5 °C after 5 min of irradiation, while the temperature of the PBS group increased by only ~2.3 °C. The photothermal conversion efficacy (*η*) of MPB-3BP@CM NPs was calculated to be ~41.08% (Supplementary Fig. 5). Besides, it was evident that despite exposure to five cycles of NIR laser irradiation, the photothermal conversion efficiency of MPB-3BP@CM NPs remained consistently high (Fig. 1m). These results confirmed the exceptional photothermal performance and stability of MPB-3BP@CM NPs, making it a promising nanoagent for PTT cancer treatment.

### Cell binding and cell uptake

Cancer cells employ various strategies to evade immune surveillance and phagocytosis, one of which involves the upregulation of CD47 ligands. These ligands interact with SIRPα receptors, transmitting a ‘don’t eat me’ signal that inhibits macrophages from engulfing the cancer cells.^28,29^ To investigate whether MPB-3BP@CM NPs could bind to CRC cells through the MSIRPα-CD47 interaction, the human CRC cancer cell lines HCT116 expressing CD47 were selected using western blot analysis (Fig. 2b), and then incubated MPB-3BP@CM NPs in vitro. To visualize the cell membrane of HCT116 cells, we utilized WGA 488 dye for staining. Simultaneously, the MSIRPα receptors fused with DsRed proteins emitted red fluorescence, serving as a fluorescent marker for MPB-3BP@CM NPs. Notably, our observations revealed robust binding of MPB-3BP@CM NPs to the cell membrane surface of HCT116 cells following a 2 h incubation period (Fig. 2a). Furthermore, to visualize the interaction between MPB-3BP@CT NPs and HCT116 cells, we employed Cy3 fluorescent labeling on the cell membrane surface of MPB-3BP@CT NPs to obtain Cy3-labeled MPB-3BP@CT NPs. In sharp contrast, Cy3-labeled MPB-3BP@CT NPs had low membrane binding affinity. To investigate whether the binding of MPB-3BP@CM NPs on the HCT116 cells was through the interaction between MSIRPα and CD47, we added a CD47 primary antibody to block the CD47 ligand on HCT116 cells. The findings demonstrated a significant reduction in the binding of MPB-3BP@CM NPs when HCT116 cells were preincubated with a CD47 primary antibody. Moreover, the molecular interaction between MSIRPα and CD47 was further detected using co-immunoprecipitation assay (CO-IP). Remarkably, CD47 was pulled down together with MSIRPα by the SIRPα primary antibody (Fig. 2c), indicating that MPB-3BP@CM NPs physically interacted with CD47 expressed on HCT116 cells. Collectively, these results confirm the effective interaction between MPB-3BP@CM NPs, presenting MSIRPα receptors on their surface, and HCT116 cells through the binding between MSIRPα and CD47.

Before MPB-3BP@CM NPs were used for therapeutic application, their cellular uptake was first evaluated in vitro. To examine the cellular uptake of MPB-3BP@CM NPs in HCT116 cells, Cy7.5 was specifically selected as the 3BP substitute due to its high fluorescence brightness and a drug loading efficiency of 7.6% in MPB-Cy7.5@CM NPs. Flow cytometry and confocal laser scanning microscopy (CLSM) were employed to analyze the HCT116 cells treated with MPB-Cy7.5@CM NPs. The flow cytometric analysis revealed that 95% of HCT116 cells were labeled with MPB-Cy7.5@CM NPs within 1 h (Fig. 2d and Supplementary Fig. 6), and the fluorescence intensity of HCT116 cells exhibited an approximately threefold increase as the coincubation time extended from 1 to 6 h (Fig. 2e). The results indicated the effective labeling of HCT116 cells with MPB-Cy7.5@CM NPs; however, the ability to differentiate between fluorescence originating from cellular uptake or binding to the cell membrane was lacking. To further validate the cellular uptake of nanoparticles, additional visualization investigations were conducted employing CLSM. As depicted in supplementary Fig. 7, the intensity of the DsRed fluorescence signal on the membrane surface increased with the extension of the coincubation time from 1 to 6 h, indicating that more nanoparticles bound around the cell membrane surface of HCT116 cells. Concomitantly, the fluorescence signal of Cy7.5 in the cytoplasm became brighter, indicating increased nanoparticle uptake. Additionally, the introducing a CD47 primary antibody to block the CD47 ligand on HCT116 cells, a substantial reduction was observed in both nanoparticle binding to the cellular membrane and internalization of nanoparticles. Finally, intracellular fluorescence signals were quantified as an indicator of uptake, and the corresponding results are presented in Fig. 2f. These collective findings indicate that HCT116 cells demonstrate a remarkable efficiency in the uptake of MPB-3BP@CM NPs.

### In vitro cytotoxicity and antitumor efficiency

Before in vivo therapy, the in vitro cytotoxicity of MPB-3BP@CM NPs was evaluated. A hemolysis test is one of the most important indexes for determining the safety of nanomedicine in vivo. A hemolytic test revealed that the MPB-3BP@CM NPs prepared did not induce hemolysis in vitro even at concentrations as high as 500 μg/mL, verifying the excellent blood compatibility of the MPB-3BP@CM NPs (Supplementary Fig. 8). Additionally, the cytotoxicity of the MPB-3BP@CM NPs was investigated using the methyl thiazolyl tetrazolium (MTT) assay. The results demonstrated negligible cytotoxic effects on HEK 293T and mouse fibroblast cell lines L929 even at concentrations up to 400 μg/mL after 6 h of coincubation period, thereby further confirming its excellent biocompatibility (Supplementary Fig. 9a).

Furthermore, the in vitro antitumor efficiency of MPB-3BP@CM NPs was evaluated using the MTT assay (Supplementary Fig. 9b). The incubation of HCT116 cells with cellular membrane nanoparticles (CM NPs or CT NPs) at various concentrations did not result in notable antitumor effects, even at elevated concentrations. However, both free 3BP, 3BP@CM NPs, and MPB-3BP@CM NPs showed dose-dependent antitumor activities. Subsequently, MPB-3BP@CM NPs were employed as a photothermal agent for in vitro cancer cell ablation upon exposure to NIR laser irradiation. The results showed MPB-3BP@CM NPs combined with laser irradiation exhibited potent anticancer activities, exhibiting significantly enhanced therapeutic efficacy compared to an equivalent dose without laser treatment and other laser groups with the same dose of photothermal agent. Collectively, the superior anticancer efficacy could be attributed to the enhanced cellular uptake of MPB-3BP@CM NPs, and chemotherapy/PTT cotherapy by MPB-3BP@CM NPs. In addition, the impact of various treatments on cell viability was assessed through live/dead cell fluorescence staining (Supplementary Fig. 10a) and apoptosis flow cytometry (Fig. 2g and Supplementary Fig. 10b), yielding results consistent with the MTT assay findings. Notably, when the concentration of MPB-3BP@CM NPs was 200 μg/mL and the concentration of other preparation groups was consistent with the content of the corresponding components in MPB-3BP@CM NPs, the killing effect of 3BP-containing preparation on HCT116 cells was significantly better than that of other preparations without 3BP. The findings suggest that 3BP-mediated inhibition of glycolysis in this nanosystem plays an important role in inducing cytotoxicity against tumor cells in vitro.

### Modulation of the cellular glycolytic pathway

Most tumor cells are recognized for their reliance on aerobic glycolysis to meet the high energy and biomass demands.^36,37^ To validate the efficacy of MPB-3BP@CM NPs in disrupting the tumor glycolytic pathway, we assessed the levels of ATP and lactate in HCT116 cells cultured for varying durations following treatment with MPB-3BP@CM NPs. This is due to the essential role of ATP in tumor cellular proliferation. Additionally, lactate, the ultimate byproduct of glycolysis, is commonly found in the tumor microenvironment (TME), where it contributes significantly to cancer progression by fostering an environment conducive to tumor pathogenesis and evolution.^34,38^ After treatment with MPB-3BP@CM NPs (200 μg/mL), HCT116 cells did not exhibit apoptosis within a short duration of time (8 h). Moreover, there was an increase in intracellular ATP levels and a decrease in intracellular lactate content (Fig. 2h). This observation is consistent with previous findings indicating that an increase in intracellular ATP levels has been observed in apoptotic cells before, which has been suggested as a requisite for apoptosis.^39^ However, following an extended period of time (14 h), the cells demonstrated evident apoptosis, accompanied by significant reductions in both ATP and lactate levels (Fig. 2i). In brief, the results indicate that MPB-3BP@CM NPs possess the ability to regulate tumor glycolytic metabolism and effectively attenuate ATP and lactate production in HCT116 cells.

In addition, glucose metabolite analysis and transcription profiling were conducted on HCT116 cells treated with MPB-3BP@CM NPs (200 µg/mL) for a duration of 8 h. This specific time point was selected to minimize potential interference arising from apoptotic processes. Both the 3BP group and the MPB-3BP@CM NPs group displayed substantial and comparable changes in cellular glucose metabolites compared to the control group (Fig. 2j and Supplementary Fig. 11a, b). The measurements results of ATP and lactate content were consistent with the previous findings, thereby validating the reliability of our results (Fig. 2k). Surprisingly, a significant accumulation of metabolites associated with the glycolysis pathway was observed, whereas a notable decrease was noted in those linked to the tricarboxylic acid cycle pathway. The presence of multiple HK isotypes allows for compensatory functioning when inhibiting the activity of HK_2_. Simultaneously, inhibition of HK_2_ activity disrupts the HK_2_-VDAC1 association, leading to impaired mitochondrial localization of HK_2_ and initiating a cascade amplification reaction of mitochondrial apoptosis,^40^ ultimately obstructing the tricarboxylic acid cycle. These reasons provide a plausible explanation for the accumulation of metabolites associated with the glycolytic pathway. Furthermore, a substantial decrease was observed in multiple cellular energy metabolism-associated substances, including adenosine diphosphate (ADP), guanosine diphosphate (GDP), nicotinamide adenine dinucleotide phosphate (NADPH). Afterward, RNA-sequence analysis revealed minimal disparity in overall gene expression between the 3BP group and the MPB-3BP@CM NPs group. Nevertheless, significant disparities were evident in overall gene expression between both treatment groups compared to the control group (Supplementary Fig. 11c, d). According to KEGG pathway analysis (Fig. 2l), the differentially expressed genes associated with metabolism were primarily enriched in starch and sucrose metabolism, inositol phosphate metabolism, glycolysis/gluconeogenesis, and galactose metabolism. The heatmap illustrated the expression levels of these representative differentially expressed genes (Fig. 2m). Here, we conducted a further analysis of specific genes that exhibited upregulation (hexokinase domain containing 1 (HKDC1) and glucokinase (GCK)) or downregulation (lactate dehydrogenase A like 6B (LDHAL6B)) in relation to the glycolysis pathway (Supplementary Fig. 11e). These findings offer further compelling evidence for discrepancies in glucose metabolites within the MPB-3BP@CM NPs group. Finally, utilizing a mouse subcutaneous HCT116 tumor model, we demonstrated the ability of MPB-3BP@CM NPs to disrupt the glucose metabolism pathway of tumor cells in vivo, leading to a significant reduction in various metabolic substances such as ATP and lactate. Moreover, this effect can be enhanced through combination with PTT (Supplementary Fig. 12). Unfortunately, no discernible impact of MPB-3BP@CM NPs on the expression of genes associated with the glycolytic pathway in tumor cells was observed in vivo. In conclusion, both in vitro and in vivo investigations have consistently demonstrated the effective regulation of tumor glucose metabolism by MPB-3BP@CM NPs.

### Induce potent immune responses

Elevated lactate levels in the TME have been associated with the promotion of angiogenesis, metastasis, and immunosuppression.^38^ Importantly, lactate accumulation in the TME correlates with infiltration and activation of TAMs. As the predominant cellular component within the TME, TAMs can impede antitumor immune responses through the secretion of diverse mediators that facilitate cancer cell proliferation and progression.^41^ Notably, the TAMs can switch to two main phenotypes: (i) the classically activated, tumor-suppressive M1 phenotype and (ii) the alternatively activated, tumor-supportive M2 phenotype.^42^ Previous studies have shown that the lactate-rich TME can educate recruited TAMs into M2-polarized TAMs.^34^ Considering the capacity of MPB-3BP@CM NPs to attenuate lactate production in HCT116 cells, we investigated whether the effective regulation of tumor glucose metabolism mediated by MPB-3BP@CMPs could facilitate polarization of TAMs towards an anti-tumorigenic M1 phenotype.

**Fig. 3:**
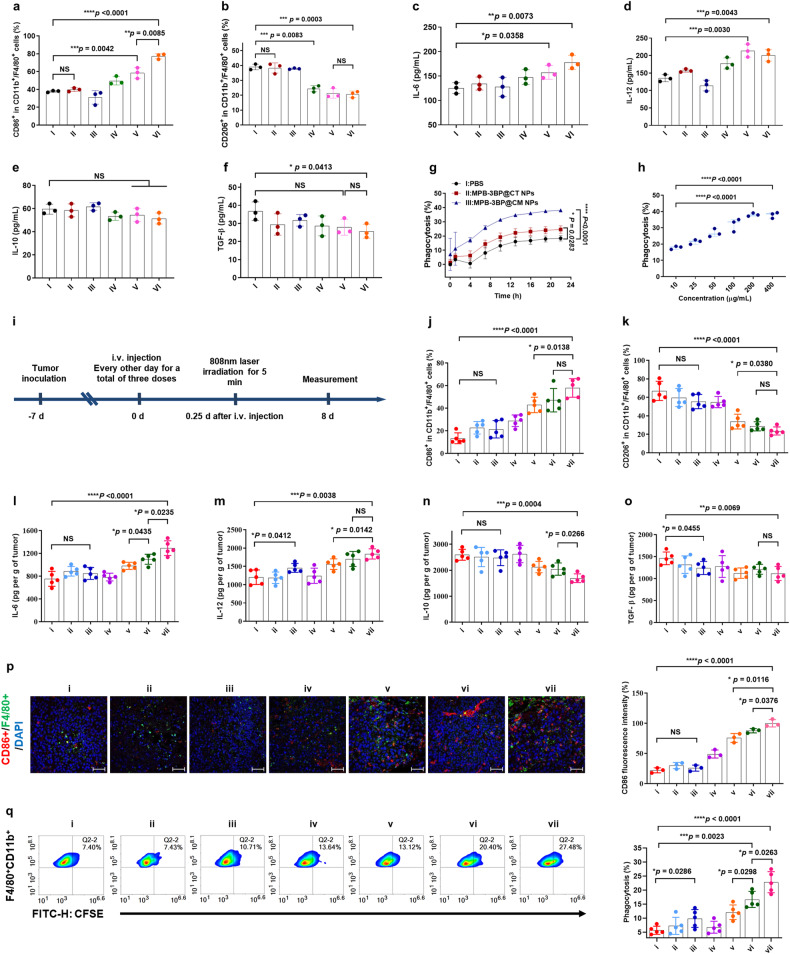
Both in vitro and in vivo, MPB-3BP@CM NPs-induced macrophage polarization and macrophage phagocytosis. The flow cytometric analysis was performed to quantify both M1-like macrophages (CD86+) (**a**) and M2-like macrophages (CD206+) (**b**) were quantified in RAW264.7 gating on F4/80 + CD11b+ cells (I: PBS, II: CM NPs, III: MPB NPs + laser, IV: 3BP, V: MPB-3BP@CM NPs, VI: MPB-3BP@CM NPs + laser). ELISA measurement of the secretion of IL-6 (**c**), IL-12 (**d**), IL-10 (**e**), and TGF-β (**f**) in different treatment groups. **g** Quantification analysis reveals that MPB-3BP@CM NPs-mediated CD47 blockade enhances phagocytosis of HCT116-EGPF cancer cells by BMDMs in vitro. **h** Quantification analysis of the phagocytosis of HCT116-EGPF cancer cells by BMDMs after CD47 blockade with varying concentrations of MPB-3BP@CM NPs in vitro. **i** Schematic illustration showing the timelines of treatment. Quantification analysis of M1-like macrophages (CD86+) (**j**) and M2-like macrophages (CD206+) (**k**) in tumor gating on F4/80 + CD11b+ cells (i: saline, ii: MPB NPs + laser, iii: 3BP, iv: CM NPs, v- MPB-3BP@CT NPs + laser, vi: MPB-3BP@CM NPs, vii: MPB-3BP@CM NPs + laser). Secretion levels of IL-6 (**l**), IL-12 (**m**), IL-10 (**n**), and TGF-*β* (**o**) in tumor tissues of mice after different treatments. **p** Representative image and quantitative analysis of immunofluorescence staining of the tumor sections showing infiltrated CD86 + F4/80+ macrophage cells. Scale bar = 50 μm. **q** Representative flow cytometry plots of CFSE + F4/80 + CD11b+ cells in tumor gating on F4/80 + CD11b+ cells, the phagocytosis efficiency is represented by the percentage of CFSE + F4/80 + CD11b+ cells in total F4/80 + CD11b+ cells. All data were presented as mean ± SD (*n* ≥ 3)

Firstly, RAW 264.7 macrophages were coculture with differentially treated HCT116 cancer cells in vitro (Supplementary Fig. 13a). Flow cytometry validated that macrophages in the MPB-3BP@CM NPs and MPB-3BP@CM NPs + laser groups, exhibited elevated levels of M1 marker CD86 and reduced levels of M2 marker CD206 compared to the control group (Fig. 3a, b and Supplementary Fig. 13b). Similar trends were observed in the 3BP group, although the effect was less pronounced compared to the MPB-3BP@CM NPs and MPB-3BP@CM NPs + laser groups. However, there was no significant difference in macrophage polarization observed between the CM NPs and MPB NPs + laser groups compared to the control group. Importantly, neither 3BP nor MPB-3BP@CM NPs exhibited direct potential in inducing M1 polarization of macrophages (Supplementary Fig. 14). Additionally, the quantification of cytokines (including IL-12 and IL-6 as markers for the M1 phenotype, TGF-*β* and IL-10 as markers for the M2 phenotype) further validated that the interference with tumor glucose metabolism mediated by MPB-3BP@CM NPs efficiently induced polarization of TAMs towards the M1 phenotype in vitro (Fig. 3c–f). Subsequently, the in vivo effects of MPB-3BP@CM NPs on efficiently polarizing TAMs towards the M1 phenotype were further investigated using an HCT116 subcutaneous tumor model in Balb/c nude mice. The tumors were collected and subjected to flow cytometry analysis. Flow cytometry analysis demonstrated that MPB-3BP@CM NPs + laser promoted the polarization of M1 macrophage (CD11b+ F4/80+ CD86+) and concomitantly inhibited the polarization of M2 macrophage (CD11b + F4/80 + CD206+) (Fig. 3j, k and Supplementary Fig. 15). Parallelly, immunofluorescence staining of tumor sections unveiled a notable rise in the count of M1 macrophages (CD86+) within the MPB-3BP@CM NPs + laser group (Fig. 3p). The increase in polarization was further validated by elevation in levels of IL-6 and IL-12 (M1 marker) and decrease in levels of TGF-β and IL-10 (M2 marker) in the tumor tissues (Fig. 3l–o), especially in the MPB-3BP@CM NPs + laser group. Flow cytometry, immunofluorescence, and cytokine results demonstrated that the polarization of TAMs towards an M1 phenotype was improved following MPB-3BP@CM NPs + laser treatment, suggesting that the enhancement of specific antitumor immunity was effectively achieved.

In addition, numerous SIRPα or CD47 blockades have shown encouraging outcomes in both preclinical studies and clinical trials.^43^ Excitingly, recent research has revealed the potential of engineered exosomes to efficiently stimulate macrophages for the phagocytosis of cancer cells.^25–27,44^ Building on this, our investigation aimed to ascertain whether MPB-3BP@CM NPs could engage with CD47 receptors on the surface of tumor cells, thus impeding the ‘don’t eat me’ signal and bolstering macrophage-mediated phagocytosis of tumor cells. In vitro, HCT116-GFP cells were selected and treated with MPB-3BP@CM NPs, followed by coculture with bone marrow-derived macrophages (BMDMs). The IncuCyte Live-cell analysis system was used for real-time monitoring of phagocytosis over 24 h. IncuCyte Live-Cell imaging analysis revealed a dose-dependent augmentation in the phagocytosis of cancer cells by BMDMs upon CD47 blockade by MPB-3BP@CM NPs (Fig. 3g, h and Supplementary Fig. 16). However, the phagocytosis of HCT116-GFP cells by BMDMs was significantly attenuated in both the MPB-3BP@CT NPs and control groups compared to the MPB-3BP@CM NPs group. Lastly, to assess the in vivo impact of MPB-3BP@CM NPs on macrophage phagocytic activity, CFSE-labeled HCT116 cells were subcutaneously engrafted into Balb/c nude mice and subjected to different treatment regimens.^45^ The tumors were collected and subjected to flow cytometry analysis. The flow cytometry results revealed a significantly elevated number of macrophages engulfing cancer cells in the MPB-3BP@CM NPs and MPB-3BP@CM NPs + laser groups, as compared to the remaining experimental groups (Fig. 3q and Supplementary Fig. 17). The results indicate that the MPB-3@CM NPs possess the ability to stimulate macrophages towards phagocytosis of tumor cells, despite the common challenge of slow diffusion of nanoparticles in tumor interstitium. Moreover, laser irradiation-mediated local transient temperature elevation of tumor tissue further enhances the phagocytic activity. In summary, both in vitro and in vivo investigations unequivocally demonstrate that MPB-3BP@CM NPs elicit a potent antitumor immune response.

### Pharmacokinetic, tissue distribution, and tumor accumulation of MPB-3BP@CM NPs

**Fig. 4:**
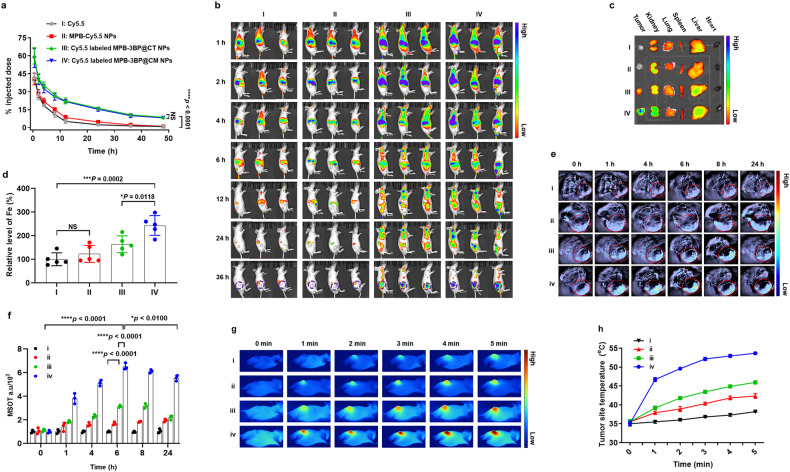
Pharmacokinetic, tissue distribution and tumor accumulation of MPB-3BP@CM NPs. **a** In vivo pharmacokinetic curves of free Cy5.5 (I), MPB-Cy5.5 NPs (II), Cy5.5-labeled MPB-3BP@CT NPs (III), or Cy5.5-labeled MPB-3BP@CM NPs (IV). **b** In vivo distribution of nanoparticles in HCT116 tumor-bearing male BALB/c nude mice examined by fluorescence imaging at predetermined time points, with the tumors demarcated by purple circles. **c** The IVIS spectrum images of the distribution of nanoparticles in tumor and major organs. **d** Quantification of nanoparticle accumulation in tumors using ICP assay. PAI (**e**) and PA intensity (MSOT a.u) (**f**) of HCT116 tumor-bearing male BALB/c nude mice taken at different time points after i.v. injection with saline (i), MPB NPs (ii), MPB-3BP@CT NPs (iii), or MPB-3BP@CM NPs (iv). The images represent the transverse sections of the mice during PAI, with the tumors demarcated by red circles. **g** IR thermographic images of HCT116 tumor-bearing male BALB/c nude mice under the 808 nm laser irradiation at the power density of 1.0 W/cm^2^ over time. **h** The temperature curves of the tumor site surface during irradiation. All data were presented as mean ± SD (*n* ≥ 3)

To assess whether MPB-3BP@CM NPs inherited a long circulation lifetime from the CM-MSIRPα, the pharmacokinetics of MPB-3BP@CM NPs was studied by using an HCT116 tumor-bearing male BALB/c nude mice model. First, we labeled MPB-3BP@CM NPs with Cy5.5. Subsequently, free Cy5.5, MPB-Cy5.5 NPs, Cy5.5-labeled MPB-3BP@CT NPs, or Cy5.5-labeled MPB-3BP@CM NPs were intravenously administered via the tail vein, and the remaining nanoparticle content was assessed by measuring the relative signal intensity of collected blood samples at specific intervals using fluorescence spectroscopy. Remarkably, Fig. 4a illustrates that ~8.2% of Cy5.5-labeled MPB-3BP@CM NPs persisted within the blood vessels even after 48 h of circulation, while both free Cy5.5 and MPB-Cy5.5 NPs had been almost completely eliminated from the bloodstream by 12 h post-injection. Interestingly, Cy7.5-labeled MPB-3BP@CT NPs and Cy7.5-labeled MPB-3BP@CM NPs had similar pharmacokinetic profiles. Collectively, the enhanced blood circulation capacity of MPB-3BP@CM NPs may stem from their “stealthy” nature, facilitated by the natural cell membrane cloaking rich in membrane proteins and antigens, thus evading detection by the host immune system. Ultimately, the extended circulation of the MPB-3BP@CM NPs is expected to sufficiently facilitate their delivery to the site of interest.

We then performed NIR fluorescence imaging to investigate the distribution within tissues and accumulation in tumors of MPB-3BP@CM NPs in the HCT116 tumor model. The fluorescence intensity in the groups treated with free Cy5.5 and MPB-Cy5.5 NPs decreased notably after 12 h, with minimal observable tumor accumulation in the mice (Fig. 4b). Conversely, in mice injected with Cy5.5-labeled MPB-3BP@CM NPs, the fluorescence signal gradually appeared and accumulated at the tumor site. This is because the inclusion of the MSIRPα receptor provided additional anchoring with CD47 ligands on tumor cells to resist diffusion. Thus, our observations revealed superior tumor site accumulation of Cy5.5-labeled MPB-3BP@CM NPs compared to Cy5.5-labeled MPB-3BP@CT NPs. After 36 h of intravenous administration, all mice were euthanized, and their main organs and tumors were harvested for ex vivo imaging (Fig. 4c). We observed a predominant accumulation of Cy5.5-labeled MPB-3BP@CM NPs in the liver, kidney, and tumor sites. The accumulation in the liver and kidney can be attributed to the unique structural and blood flow features of these organs, which facilitate nanoparticle sequestration.^46,47^ Additionally, MPB-3BP@CM NPs exhibit a high affinity to CD47-expressing normal tissue cells owing to the presence of MSIRPα on their surface. However, it is noteworthy that Cy5.5-labeled MPB-3BP@CM NPs exhibited the highest tumor accumulation compared to the other groups. The quantitative assessment of nanoparticle accumulation in tumor tissues was further conducted using an inductively coupled plasma emission spectrometer (ICP) assay, with Fe serving as the indicator. The results revealed a significantly higher amount of Fe in the tumor tissues of the Cy5.5-labeled MPB-3BP@CM NPs group compared to the other groups (Fig. 4d). Furthermore, we conducted an in vivo PAI study to investigate the effective accumulation of MPB-3BP@CM NPs in tumors. The results demonstrated excellent tumor targeting, with the maximum accumulation reached at 6 h (Fig. 4e, f). In addition, because of their ability to achieve the desired tumor targeting and accumulation, MPB-3BP@CM NPs showed high photothermal conversion efficiency in vivo, with the tumor temperature in the MPB-3BP@CM NPs + laser group escalated to 53.7 °C after 5 min upon laser radiation (Fig. 4g, h). To summarize, these data provide compelling evidence that CM-MSIRPα-coated MPB-3BP@CM NPs possess remarkable long circulation capacity, sufficient to reach the target tissue, underlining the advantages of MPB-3BP@CM NPs for tumor targeting and drug delivery.

### Antitumor in vivo

**Fig. 5:**
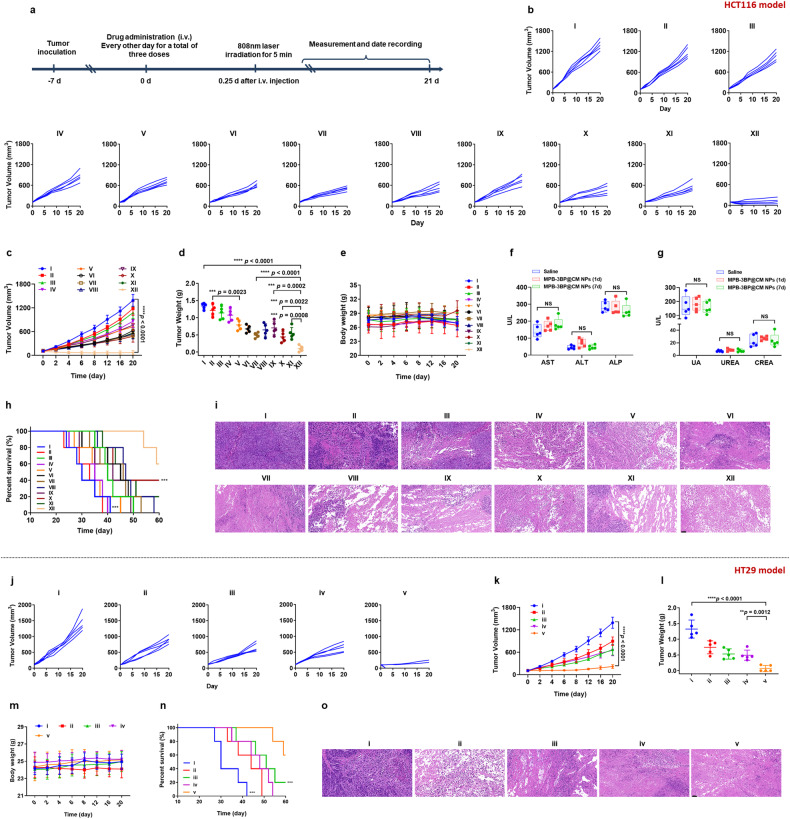
The in vivo anticancer efficacy of MPB-3BP@CM NPs. Firstly, the in vivo anticancer efficacy of MPB-3BP@CM NPs was investigated using an HCT116 tumor model. **a** Schematic illustration showing the timelines of treatment. Individual (**b**) and average (**c**) tumor growth curves of various groups (I: saline, II: CT NPs, III: MPB NPs + laser, IV: 3BP, V: CM NPs, VI: MPB-3BP NPs + laser, VII: MPB@CM NPs + laser, VIII: 3BP@CM NPs, IX: MPB NPs/3BP/CM NPs + laser, X: MPB-3BP@CT NPs + laser, XI: MPB-3BP@CM NPs, and XII: MPB-3BP@CM NPs + laser). **d** Tumor weight for different treatment groups. **e** The body weight of the mice in each group. Serum levels of hepatic (**f**) and kidney (**g**) functional biomarkers. **h** Survival curves for different treatment groups. **i** Representative H&E staining of tumor in each group. Then, the in vivo anticancer efficacy of MPB-3BP@CM NPs was investigated using an HT29 tumor model. Individual (**j**) and average (**k**) tumor growth curves of various groups (i: saline, ii: MPB NPs/3BP/CM NPs + laser, iii: MPB-3BP@CT NPs + laser, iv: MPB-3BP@CM NPs, and v: MPB-3BP@CM NPs + laser). **l** Tumor weight for different treatment groups. **m** The body weight of the mice in each group. **n** Survival curves for different treatment groups. **o** Representative H&E staining of tumor in each group. Scale bar = 50 μm. All data were presented as mean ± SD (*n* = 5)

Building upon the established efficacy of MPB-3BP@CM NPs in disrupting tumor glucose metabolism, stimulating tumor immunity, and exhibiting remarkable tumor targeting capabilities, we conducted further investigations into the anti-CRC efficacy of MPB-3BP@CM NPs. Firstly, the anticancer performance of MPB-3BP@CM NPs in vivo was investigated using the HCT116 tumor model. The mice were monitored until the tumor volume reached ~100 mm^3^, and then randomly divided into 12 groups for various treatment regimens (Fig. 5a). Tumor growth inhibition was observed in groups treated with 3BP, MPB NPs + laser, and MPB-3BP NPs + laser, exhibiting inhibition rates of 80.0, 84.8, and 50.2%, respectively, compared to the saline group (Fig. 5b–d). This inhibition can be attributed to the passive accumulation effect of the drug at tumor sites. Notably, the tumor inhibition rate of 3BP@CM NPs, MPB@CM NPs + laser, and MPB-3BP@CM NPs+ laser coated with the membrane was 31.9.1, 48.0, and 42.7% higher compared with that of 3BP alone, MPB NPs + laser and MPB-3BP NPs + laser group, respectively. This improvement in tumor inhibition rate was attributed to CM-MSIRPα endowed 3BP@CM NPs, MPB@CM NPs, and MPB-3BP@CM NPs with the capacity to remain stable in the blood circulation for a prolonged time in vivo. Furthermore, these modifications facilitated precise tumor targeting and improved the release of 3BP and PB NPs for effective suppression of tumor growth. Based on similar principles, the tumor inhibition rate of the MPB-3BP@CM NPs + laser group was significantly improved compared with the MPB-3BP@CT NPs + laser group. Moreover, unlike liposomes and other cell membrane nanocarriers, CM-MSIRPα contains the transmembrane protein MSIRPα that blocks CD47-SIRPα interaction and promotes macrophage phagocytosis. Therefore, the tumor inhibition rate of the CM NPs group was increased by 31.6% compared with the CT NPs group. Furthermore, PTT mediated by MPB NPs had a pivotal role in the nanosystem. Notably, in the MPB-3BP@CM NPs + laser group, the synergistic effect of photothermal treatment markedly augmented tumor inhibition compared to the MPB-3BP@CM NPs group. To further elucidate the therapeutic efficacy, hematoxylin and eosin (H&E), Ki67, and terminal-deoxynucleotidyl transferase-mediated nick end labeling (TUNEL) assays were conducted to evaluate apoptosis and proliferation levels within the tumor tissue. Remarkably, the MPB-3BP@CM NPs + laser group exhibited heightened tumor cell necrosis and apoptosis relative to the other treatment groups (Fig. 5i and Supplementary Fig. 18). Additionally, assessment of body weight and organ histology across the various treatment groups revealed no statistically significant differences in weight or histopathological abnormalities, underscoring the safety and biocompatibility of the administered treatments (Fig. 5e and Supplementary Fig. 19). Furthermore, the results of blood routine and blood biochemistry analysis further confirmed the biocompatibility and safety of MPB-3BP@CM NPs (Fig. 5f, g and Supplementary Table 2). Lastly, we monitored the survival duration of mice following different treatment regimens. Notably, the MPB-3BP@CM NPs + laser group exhibited a significantly prolonged survival rate (Fig. 5h).

Furthermore, the human colon cancer cell line HT29 was selected for further investigation to provide additional evidence supporting the anti-CRC efficacy of MPB-3BP@CM NPs. In vitro, the combination of MPB-3BP@CM NPs and laser irradiation can effectively suppress the proliferation and induce apoptosis of HT29 cells (Supplementary Fig. 20). Consistently, this combination demonstrates potent inhibitory effects on HT29 tumor growth in vivo, significantly prolonging mouse survival (Fig. 5j–n and Supplementary Fig. 21a). Moreover, the findings of H&E, Ki67, and TUNEL assays of tumor tissues provided compelling evidence that MPB-3BP@CM NPs combined with laser irradiation induced significantly more necrosis and apoptosis in tumor cells compared to other treatment groups (Fig. 5o and Supplementary Fig. 21b, c). Additionally, no significant alterations in body weight were noted across all experimental groups throughout the monitoring period, thereby providing further evidence to substantiate the biocompatibility and safety of MPB-3BP@CM NPs. Collectively, these observations have demonstrated the favorable efficacy and safety profile of MPB-3BP@CM NPs for CRC treatment.

## Discussion

In recent years, metabolic research has been a prominent topic in the field of oncology. The unique metabolic pattern of tumor cells endows the tumor cells with malignant biological characteristics. Among these, heightened glycolysis, known as the Warburg effect, stands out as a hallmark feature, driven by dysregulated glycolytic enzymes. Targeting key enzymes involved in glycolysis, such as the inhibition of enzymes glucose transporter 1 (GLUT1), HK_2_, pyruvate kinase M2 (PKM2), and lactate dehydrogenase (LDH), can achieve a potent strategy to inhibit tumor proliferation, invasion and metastasis, immune evasion, angiogenesis, and drug resistance.^48,49^ HK_2_ plays a pivotal role as a rate-limiting enzyme in the aerobic glycolysis pathway, directly regulating the amount of glucose entering glycolysis. While the HK_2_ inhibitor, 3BP, shows promise in combating tumors, its free form grapples with instability during systemic administration due to its alkylating nature. To tackle this challenge, we utilized MPB NPs for encapsulation, effectively improving its stability. Simultaneously, at the core of tumor development lies the intricate interplay within the TME, a dynamic ecosystem comprising a diversity of cell populations, both tumorous and non-tumorous. Each cellular component within this milieu presents distinct metabolic profiles, contributing to the overall metabolic landscape of the tumor.^50^ Therefore, the therapeutic potential of glycolysis inhibitors is limited, and thus the combination of glycolytic inhibitors with other anticancer therapies may provide a more effective and comprehensive approach to tumor treatment. PTT utilizes photothermal agents to convert NIR photoirradiation to localized hyperthermia for tumor ablation. In addition, the heat generated during PTT process can control the release of therapeutic agents, trigger antitumor immune response, regulate intracellular gene expression and enzyme activity.^18^ Prussian blue, FDA-approved for treating radioactive cesium and thallium poisoning, demonstrates remarkable biocompatibility. Continued investigation into Prussian blue nanoparticles highlights their potential as a promising PTT and drug delivery platform, capturing significant interest within the biomedical field. Therefore, the combination of the HK_2_ inhibitor 3BP with MPB NPs-mediated PTT may yield substantial therapeutic efficacy against CRC.

However, the precise delivery of nanomedicines to tumor sites remains an intractable clinical puzzle. Acknowledging the vital role of the biological interface of nanoparticles, intentional surface engineering has become recognized as crucial in the design process. Thus, driven by insights from biotechnology, researchers have ventured into creating biomimetic nanoparticles that mimic cell membrane properties. This innovation has given rise to a novel category of nanoparticles known as cell membrane-coated biomimetic nanoparticles, which combine the advantages of natural and artificial nanomaterials while mimicking the properties of their source cells. The membranes endow the nanoparticles with unique properties such as immune escaping, long circulation, and disease-relevant targeting, which can be exploited to promote precise delivery of nanomedicines to tumor sites.^41–43^ Nevertheless, natural cell membranes are inadequate for clinical applications due to resource scarcity and functional limitations. To address this issue, it is imperative to engineer customized cell membranes through genetic editing. This approach not only broadens the spectrum of potential nanoformulations but also transcends the capabilities achievable with natural cell membranes. By applying this approach universally, researchers can expedite the creation of targeted nanoformulations, leveraging target-ligand interactions found in nature. Overall, the integration of cell membrane coating technology and genetic engineering is anticipated to spark transformative breakthroughs in upcoming therapeutic developments.

In summary, we have successfully constructed a hybrid nanoparticle MPB-3BP@CM NPs using a cell membrane overexpressing high-affinity SIRPα variants, which was coated on MPB NPs loaded with 3BP. Compared to other synthetic nanocarriers, MPB-3BP@CM NPs inherit the features of the cell membrane and show high stability, biocompatibility, prolonged circulation time, and improved tumor accumulation. As MPB-3BP@CM NPs accumulate in tumor tissues, they enhance macrophage phagocytosis of tumor cells by competitively blocking the SIRPα-CD47 interaction between tumor cells and macrophages. In addition, MPB-3BP@CM NPs block ATP supply and reduce tumor lactate production by releasing 3BP to inhibit glycolysis, leading to starvation-induced apoptosis and modulating the TME to inhibit tumor progression. Besides, MPB-3BP@CM NPs can repolarize TAMs towards the M1 phenotype, thereby inducing intense immune responses to kill tumor cells. Moreover, the utilization of NIR photoirradiation in combination with MPB-3BP@CM NPs can serve as a PTT agent to augment tumor therapy. The aforementioned benefits render MPB-3BP@CM NPs a promising multimodal agent for CRC theranostics, as evidenced by a significant delay in tumor growth and prolonged survival time, without observable systemic toxicity. Future investigations will comprehensively assess the safety of the MPB-3BP@CM NPs formulation, gather further data on its efficacy against CRC, determine the optimal treatment time window, and evaluate clinical relevance through the utilization of diverse CRC animal models, including those pertaining to CRC metastasis.

The combination of glycolysis inhibitors with PTT exhibits potent anticancer effects in two subcutaneous CRC tumor models, as demonstrated in our study. Therefore, targeting the inhibition of the glycolytic pathway, along with leveraging the advantages of PTT, such as its low invasiveness and precise spatiotemporal control, shows tremendous potential in CRC therapy. Moreover, this combined treatment approach may have broader applicability to various tumor therapies. In addition, it is worth noting that the clinical utilization of genetically engineered cellular platforms has witnessed a significant surge in recent years. A pivotal moment occurred in 2017 with the FDA’s approval of CAR-T cell therapy for B-cell lymphoma, marking a significant milestone in the field.^51^ Since then, CAR-T cell therapy has been expanding applications in treating other cancers, such as solid tumors expressing epithelial cellular adhesion molecules, natural killer group 2D-ligands, mesothelin, carcinoembryonic antigen, and epidermal growth factor receptors.^52,53^ With the evolution of these trends, engineered cell nanoparticles offer exceptional design flexibility, enhanced functionality, and exceptional biocompatibility, providing an appealing prospect for addressing the most critical challenges in the field of biomedicine. Consequently, customized cell membrane preparation tailored to the unique physiological and structural characteristics of various tumor diseases has become feasible. In brief, the individually customized cell membranes offer a high degree of freedom in programmable synthesis, and is likely to promote the field of personalized medicine.

Taken together, our research not only presents a straightforward approach to CRC therapy but also leverages the inherent biocompatibility and biointerfacing properties of cell membrane coating to provide a universal strategy for developing disease-customized cell membranes for tumor treatment.

## Materials and methods

### Materials

Hygromycin B was purchased from BioFroxx Co., Ltd. NHS-Cy3 and NHS-Cy5.5 were purchased from Beijing Fanbo Biochemicals Co., Ltd. Enhanced ATP Assay Kit and CO-IP were purchased from Beyotime Biotechnology Co., Ltd. Lactate Assay Kit was purchased from Solarbio Science Technology Co., Ltd. Granulocyte-macrophage colony-stimulating factor (GM-CSF) (91108ES08) and Plasmid Preparation Kit (19036ES10) were purchased from Shanghai Yeasen Biotechnology Co., Ltd. Anti-CD47 antibody and anti-SIRPα antibody were purchased from Abcam Co., Ltd. CD86-APC monoclonal antibody, CD206-PE-cyanine 7 monoclonal antibody, CD11b-FITC monoclonal antibody, F4/80-PE monoclonal antibody, F4/80-PE-cyanine 7 monoclonal antibody, CD11b-APC monoclonal antibody, CellTrace™ CFSE, and WGA 488 were purchased from Thermo Fisher Scientific Co., Ltd. Human CRC cancer cell lines HCT116 and HT29, human embryonic kidney cell lines 293 T were all purchased from Chinese Academy of Sciences Cells Bank (Shanghai, China). Unless stated otherwise, all chemicals were obtained from Sigma-Aldrich.

### Mice

Male BALB/c nude mice (5−6 weeks old) and male C57BL/6 (5−6 weeks old) were both obtained from Nanjing GemPharmatech Laboratory Animal Technology Co., Ltd. All animal procedures were conducted in accordance with the guidelines provided by the animal care and use committee of the State Key Laboratory of Biotherapy, West China Hospital, Sichuan University.

### Plasmid construction and cell lines

The sequence of MSIRPα was synthesized and cloned in-frame into pCMV3 mammalian expression plasmid with a carboxyl-terminal DsRed tag to construct the expression plasmid (pCMV3-MSIRPα-DsRed). To construct HEK 293T-MSIRPα cells, transient transfection of HEK 293T cells was performed using linearized plasmids (pCMV3-MSIRPα-DsRed was digested by Bgl II) with lipofectamine 3000 (Invitrogen), and further selected with hygromycin B to obtain HEK 293T cells stably expressing MSIRPα. BMDMs were obtained by isolating bone marrow cells from the femur and tibiae of C57BL/6 mice, followed by differentiation using GM-CSF.

### Cell membrane derivation

The HEK 293T-MSIRPα cells were harvested and suspended in hypotonic lysing buffer (protease inhibitor cocktail, 1.0 mM EDTA, 0.25 M sucrose, 20.0 mM Hepes, and pH 7.4), and disrupted by a Dounce homogenizer with a tight-fitting pestle. Subsequently, the solution underwent centrifugation at 1000 × *g* for 5 min. The resulting supernatant was collected and underwent further centrifugation at 10,000 × *g* for 20 min, following which the pellet was discarded. The supernatant underwent another centrifugation at 100,000 × *g* for 1 h. The resulting pellet was then washed three times with PBS buffer containing protease inhibitors. The final pellet was collected and utilized as a purified cell membrane (CM-MSIRPα) for subsequent experiments. The cell membranes from HEK 293T cells were successfully isolated using the same methodology.

### Preparation and characterization of MPB-3BP@CM NPs

The synthesis of MPB NPs followed previously published protocols.^21^ MPB NPs (2.0 mg) was dispersed in 3BP solution (4.0 mL, 20.0 mg/mL), vacuum ultrasound for 30 min, then the mixture was stirred for 4 h, followed by centrifugation to collect nanoparticles. These nanoparticles were then added to the CM-MSIRPα membrane solution (2.0 mL, 300.0 µg/mL, protein weight) with sonication and subjected to continuous sonication for an additional 5 min. The resulting mixture was extruded through 400-nm and 200-nm polycarbonate porous membranes using a mini extruder from Avanti Polar Lipids, followed by centrifugation and washing with PBS buffer to collect MPB-3BP@CM NPs. The same way, MPB-3BP NPs coated with cell membrane derived from HEK 293T cells (MPB-3BP@CT NPs), HEK 293T cell membrane nanoparticles (CT NPs), CM-MSIRPα nanoparticles (CM NPs), CM-MSIRPα camouflaged MPB NPs (MPB@CM NPs), and 3BP-loaded CM-MSIRPα nanoparticles (3BP@CM NPs, with an approximately 20% loading of 3BP) were prepared.

DLS technique using Nano-ZS 90 instrument from Malvern Instruments, UK, was employed to determine the particle size and zeta potential of MPB-3BP@CM NPs. The hydrodynamic size distribution was determined based on the intensity particle size distribution (PSD). Additionally, the micromorphology of MPB-3BP@CM NPs was investigated using TEM with Tecnai G2 F20 S-TWIN. The membrane proteins were characterized through SDS-PAGE and western blot analysis. Moreover, an in vitro hemolytic study was conducted following our previously established protocols.^17^

### IP assay

To detect the orientation of MSIRPα receptors on MPB-3BP@CM NPs, the IP assay was performed. Briefly, protein A/G beads were preincubated with 1 mL (2.0 mg/mL) of MPB-3BP@CM NPs for 2 h at room temperature to remove nonspecifically bound proteins. The supernatant was collected and incubated overnight with 5 µg SIRPα primary antibody at 4 °C, and incubated with 5 µg anti-IgG as a negative control antibody. Following this, 50 µL of protein A/G-agarose beads were introduced and incubated for 3 h at room temperature. The beads underwent gentle washing with PBS buffer five times. Subsequently, the agarose beads underwent boiling in SDS sample buffer for 5 min, followed by centrifugation. The resulting samples were separated on a 10% SDS-PAGE gel in preparation for western blotting using the specified antibodies.

### In vitro release kinetics of MPB-3BP@CM NPs

The MPB-3BP@CM NPs (20.0 mg) were dispersed in 2.0 mL of PBS buffer and subsequently transferred to a dialysis bag with a molecular weight cutoff of 3500 Da. The bag was submerged in 5.0 mL of PBS buffer at 4 °C. Periodically, 0.5 mL of the medium was withdrawn and replaced with an equal volume of PBS buffer. After 48 h, the dialysis bag containing MPB-3BP@CM NPs was removed and subjected to irradiation using an 808 nm laser with a power density of 1.0 W/cm^2^ for 5 min. The concentration of 3BP was measured by high-performance liquid chromatography (HPLC). Solvent A (6% methanol containing 0.1% acetic acid) and solvent B (94% water containing 0.1% acetic acid) were used for isocratic elution at a flow rate of 1.0 mL/min. Detection was carried out at a wavelength of 208 nm employing a diode array detector (DAD).

### Photothermal performance of MPB-3BP@CM NPs

To evaluate the photothermal effect of MPB-3BP@CM NPs, solutions with varying concentrations of MPB-3BP@CM NPs were prepared. The 808 nm NIR laser beam, operating at a power density of 1.0 W/cm^2^, was directed through a 1.5 mL EP tube containing a 1.0 mL aqueous dispersion of samples. Temperature measurements were conducted using a Fluke infrared thermal imaging system (Ti-32, Fluke). The photothermal conversion efficiency (*η*) was calculated following our previous methodology.^17^ To assess the stability of the photothermal conversion of MPB-3BP@CM NPs, a solution of 0.5 mg/mL MPB-3BP@CM NPs was prepared and exposed to an 808 nm laser (1.0 W/cm^2^) for 5 min before being turned off. The laser was reactivated after a 5-min interval. This cycle was repeated five times, and temperature changes were recorded. All experiments were conducted in triplicate.

### Nanoparticle cell binding assay

Firstly, NHS-Cy3 labeling was performed on MPB-3BP@CT NPs in PBS buffer, followed by an overnight incubation at 4 °C. Post-incubation, the nanoparticles were subjected to three washes with PBS buffer to yield Cy3-labeled MPB-3BP@CT NPs. To assess the binding efficacy of MPB-3BP@CM NPs with HCT116 cancer cells, HCT116 cells were seeded in the confocal dishes. Subsequently, HCT116 cells were incubated with either MPB-3BP@CM NPs (200 µg/mL) or Cy3-labeled MPB-3BP@CT NPs (200 µg/mL) for 2 h. Alternatively, HCT116 cells were preincubated with an anti-CD47 antibody for 2 h prior to the addition of MPB-3BP@CM NPs in the culture medium. Afterward, the cells were rinsed with cold PBS buffer at least three times. Subsequently, WGA 488 was introduced to label the cell membranes for 10 min, followed by staining of the nuclei with DAPI for another 10 min. Finally, fluorescence images of the HCT116 cells were captured using CLSM. Additionally, CO-IP assays were conducted to investigate the interaction between MSIRPα on MPB-3BP@CM NPs and CD47 on HCT116 cells.

### Cellular uptake

To assess the cellular uptake of HCT116 to MPB-3BP@CM NPs, Cy7.5 with high fluorescence brightness and an encapsulation efficiency at 7.6% was specifically chosen as a substitute for 3BP in this study. The preparation method for MPB-Cy7.5@CM NPs is identical to that of MPB-3BP@CM NPs. HCT116 cells were plated onto confocal dishes, and MPB-Cy7.5@CM NPs (200 µg/mL) were introduced to the dishes for incubation with the cells for varying periods. Alternatively, an anti-CD47 antibody were preincubated with HCT116 cells for 2 h before adding MPB-Cy7.5@CM NPs to the culture medium. Following incubation for various durations, the cells underwent washing with cold PBS buffer on more than three occasions. Subsequently, WGA 488 conjugate was applied to label the cell membranes for 10 min, followed by DAPI staining of the nuclei for another 10 min. Post-staining, the cells were washed thrice with PBS buffer. Finally, fluorescence images of the cells were captured using CLSM. Additionally, a flow cytometry assay was employed to determine the cellular internalization of HCT116 to MPB-Cy7.5@CM NPs. All experiments were performed in triplicate.

### In vitro cytotoxicity assay and antitumor assay

HCT116, HT29, HEK 293T, and L929 cells were seeded into 96-well plates and allowed to grow to ~75% confluence at 37 °C with 5% CO_2_. Subsequently, the culture medium was replaced with fresh medium containing various concentrations of 3BP, CT NPs, CM NPs, MPB NPs, 3BP@CM NPs, MPB-3BP NPs, MPB@CM NPs, MPB NPs/3BP/CM NPs (5:1:4, m/m/m), MPB-3BP@CT NPs, or MPB-3BP@CM NPs was added into the corresponding plates. After 6 h of incubation, the culture medium of each sample was refreshed following a single rinse with PBS buffer, and the cells were allowed to culture for an additional 20 h. For groups subjected to NIR laser treatment, after the addition of fresh medium, the cells were exposed to an 808 nm laser at a power density of 1.0 W/cm^2^ for 5 min before another 20 h of incubation. Finally, cell viability was assessed using an MTT assay.

Further experimental groups were established utilizing various formulations, and cells were subjected to the aforementioned treatment protocols to assess their impact on both cell apoptosis and necrosis. The formulations included: 3BP (20.0 μg/mL), CT NPs (80.0 μg/mL), CM NPs (80.0 μg/mL), MPB NPs (100.0 μg/mL), 3BP@CM NPs (100.0 μg/mL), MPB-3BP NPs (120.0 μg/mL), MPB@CM NPs (180.0 μg/mL), MPB NPs/3BP/CM NPs (5:1:4, m/m/m, 200.0 μg/mL), MPB-3BP@CT NPs (200.0 μg/mL), or MPB-3BP@CM NPs (200.0 μg/mL). All experiments were performed in triplicate.

### Measurement of intracellular ATP and lactate levels

HCT116 cells were cultured in a six-well plate and preincubated at 37 °C with 5% CO_2_. Upon reaching approximately 75% confluence, the growth medium was aspirated, and fresh medium containing either 3BP (20.0 μg/mL) or MPB-3BP@CM NPs (200.0 μg/mL) was added to the respective wells. After 6 h of incubation, the culture medium of each sample was refreshed following a single rinse with PBS buffer, and the cells were maintained under standard culture conditions. Subsequently, cells were harvested at different time points for apoptosis detection, while intracellular levels of ATP and lactate were quantified. All experiments were performed in triplicate.

### RNA-Seq and metabolic assays

HCT116 cells were cultured in a 10-cm diameter petri dish and preincubated at 37 °C with 5% CO_2_. The subsequent experimental procedures follow the same operational steps as those of the measurement of intracellular ATP and lactate level assay. After a duration of 8 h, the cells were subsequently collected by rinsing them three times with PBS buffer. The collected cell samples were dispatched to Shanghai Bioprofile Technology Co., Ltd. for liquid chromatograph-mass spectrometer (LC-MS) analysis.

In a similar manner, cellular samples subjected to various treatments were collected and processed with a Trizol reagent to extract total RNA. Then the samples were sequenced using the NovaSeq 6000 platform (Illumina) at Shanghai Personal Biotechnology Cp. Ltd. Reference genome and gene annotation files were obtained from the genome website. The filtered reads were aligned to the reference genome using HISAT2 (v2.1.0). HTSeq (v0.9.1) was employed to calculate the Read Count values for each gene, serving as the baseline gene expression. The expression values were then normalized using FPKM. Differential gene expression analysis was conducted with DESeq (v1.38.3) under the following criteria: expression difference multiple |log2FoldChange| >1, significant *P* value < 0.05. Heatmaps were generated based on gene expression levels across different samples and the expression patterns of different genes within the same sample, utilizing the Euclidean distance and Complete Linkage method for clustering. GO enrichment analysis of differential genes (all DEGs/up DEGs/down DEGs) was performed using topGO (v2.50.0), with *P* values calculated via the hypergeometric distribution method (significant enrichment defined as *P* value < 0.05). Additionally, ClusterProfiler (v4.6.0) software was utilized to explore the enrichment of KEGG pathways among the differential genes, focusing on pathways with a significant enrichment (*P* value < 0.05). The data analysis was conducted using the free online platform Personalbio GenesCloud (https://www.genescloud.cn).

### In vitro macrophage polarization evaluation

Macrophage polarization assays were performed using a dual-chamber transwell system with a 0.4-µm-sized microporous membrane (Corning, USA). Briefly, HCT116 cells were seeded in the lower chamber and preincubated at 37 °C with 5% CO_2_. Upon reaching ~75% confluence, the initial medium was aspirated, and fresh medium containing different nanoparticles was added into the corresponding lower chamber (I: PBS, II: CM NPs (80.0 μg/mL), III: MPB NPs + laser (100.0 μg/mL), IV: 3BP (20.0 μg/mL), V: MPB-3BP@CM NPs (200.0 μg/mL), VI: MPB-3BP@CM NPs + laser (200.0 μg/mL)). After 6 h of incubation, the culture medium of each sample was refreshed following a single rinse with PBS buffer. For the groups subjected to laser treatment (808 nm, 5 min, 1.0 W/cm^2^), the laser was introduced after the addition of a fresh culture medium. Then, 1 × 10^6^ RAW264.7 macrophages were seeded in the upper chamber. Following 48 h of incubation, RAW264.7 macrophages from the upper chamber were acquired and stained with fluorescence-conjugated anti-mouse CD11b (eBioscience, FITC), CD206 (eBioscience, PE-cyanine 7), CD86 (eBioscience, APC), F4/80 (eBioscience, PE) antibodies. The samples were subjected to analysis using a Beckman Coulter flow cytometer. All experiments were performed in triplicate.

### In vitro macrophage phagocytosis evaluation

HCT116-EGPF cancer cells were plated in six-well plates and preincubated at 37 °C with 5% CO_2_ until reaching ~75% confluence. The cells exposed to PBS, MPB-3BP@CT NPs (200 µg/mL), or MPB-3BP@CM NPs (200 µg/mL) for a duration of 2 h. Following treatment, the HCT116-EGPF cells were harvested and thoroughly washed with PBS buffer on multiple occasions. Subsequently, 4 × 10^5^ of treated HCT116-EGPF cells were co-cultured with pre-seeded BMDMs in 24-well plates. Real-time analysis of phagocytosis of HCT116-EGPF cells by macrophages was analyzed by IncuCyte. All experiments were performed in triplicate.

### Tumor model

HCT116 subcutaneous tumors were induced by injecting 5 × 10^6^ cells suspended in approximately 100 μL serum-free McCoy’s 5A medium into the back of each male BALB/c nude mouse. Similarly, subcutaneous transplantation models of CFSE-labeled HCT116 and HT29 tumors were established.

### In vivo pharmacokinetics and biodistribution

To investigate the in vivo pharmacokinetics of MPB-3BP@CM NPs, both MPB-3BP@CM NPs and MPB-3BP@CT NPs were labeled with NHS-Cy5.5 in PBS buffer and then allowed to incubate overnight at 4 °C. Then the mice were i.v. injected with 200 μL of Cy5.5, MPB-Cy5.5 NPs, Cy5.5-labeled MPB-3BP@CT NPs, or Cy5.5-labeled MPB-3BP@CM NPs (Each containing 10 μg Cy5.5 per mouse). Blood samples were collected at predetermined time intervals and subsequently centrifuged at 3000×*g* for 10 min. The resulting supernatant was collected to measure the fluorescence intensity of the nanoparticles using a microplate reader.

To investigate the in vivo biodistribution of MPB-3BP@CM NPs, an HCT116 tumor model was established in male BALB/c nude mice. Once the tumor volumes reached approximately 200 mm^3^, the mice were i.v. injected with 200 μL of free Cy5.5, MPB-Cy5.5 NPs, Cy5.5-labeled MPB-3BP@CT NPs, or Cy5.5-labeled MPB-3BP@CM NPs (Each containing 10 μg Cy5.5 per mouse). Fluorescence images of mice at 1, 2, 4, 6, 12, 24, and 36 h were obtained via an in vivo imaging system (IVIS Spectrum, PerkinElmer). At 36 h post-injection, tumor tissues and major organs were collected for fluorescence imaging and ICP assay. In addition, PAI imaging was conducted following previously described methods.^17^

### In vivo photothermal conversion

To evaluate photothermal conversion, we established an HCT116 tumor model in male BALB/c nude mice. When the tumor volumes reached approximately 100 mm^3^, mice were i.v. injected with 200 μL of saline, MPB NPs (15.0 mg/kg), MPB-3BP@CT NPs (30.0 mg/kg), or MPB-3BP@CM NPs (30.0 mg/kg). After 6 h post-injection, mice were anesthetized with isoflurane, and their tumor areas were exposed to an 808 nm laser at a power density of 1.0 W/cm^2^ for 5 min. Using the Fluke infrared thermal imaging system, we recorded and compared the temperature of the tumor site before and after laser irradiation.

### Evaluation of in vivo immune responses

The HCT116 tumor model was established in male BALB/c nude mice. Once the tumor volumes reached ~200 mm^3^, the tumor-bearing mice were randomly allocated into three groups (I: saline, II: MPB-3BP@CM NPs (30.0 mg/kg), III: MPB-3BP@CM NPs + laser (30.0 mg/kg)). Subsequently, each mouse from the different experimental groups was i.v. injected with 200 μL of the specified drug, with administration taking place every other day for a cumulative total of three doses. In the laser treatment groups, tumors received exposure to an 808 nm laser operating at a power density of 1.0 W/cm^2^ for 5 min following intravenous injection 6 h prior. The tumor was harvested on the 8th day post-initial injection, and then the tumor tissue was dissected into small pieces. A subset of tumor samples was dispatched to Shanghai Bioprofile Technology Co., Ltd. for LC-MS analysis, while another subset underwent sequencing by Novogene Co., Ltd.

#### In vivo macrophage polarization evaluation

Seven experimental groups (i: saline, ii: MPB NPs + laser (15.0 mg/kg), iii: 3BP (3.0 mg/kg), iv: CM NPs (12.0 mg/kg), v: MPB-3BP@CT NPs + laser (30.0 mg/kg), vi: MPB-3BP@CM NPs (30.0 mg/kg), vii: MPB-3BP@CM NPs + laser (30.0 mg/kg), *n* = 5 for each group) were established, and tumor samples were collected on day 8 using the identical treatment regimen as previously described. A portion of the tumors was sectioned into small fragments and enzymatically digested in RPMI medium supplemented with 0.2 mg/mL DNases, 1.0 mg/mL collagenase I, and 0.1 mg/mL hyaluronidase at 37 °C for 2 h. The resulting mixture was filtered through 70-μm cell strainers to obtain a single-cell suspension. Subsequently, the single-cell suspensions were stained with fluorescence-conjugated anti-mouse CD11b (eBioscience, FITC), CD206 (eBioscience, PE-cyanine 7), CD86 (eBioscience, APC), F4/80 (eBioscience, PE) antibodies, followed by analysis using Beckman coulter flow cytometry. Another portion of tumors was homogenized in cold PBS buffer, followed by detection of cytokine levels according to the measurement manual. Furthermore, the remaining tumors were fixed in 4% neutral buffered formalin, followed by standard paraffin embedding and sectioning procedures. Immunofluorescence staining was performed to investigate TAMs which were then examined using CLSM.

#### In vivo macrophages phagocytosis evaluation

The CFSE-labeled HCT116 subcutaneous transplanted tumor model was adopted. An identical experimental group was established based on the in vivo evaluation of macrophages polarization, and the same treatment regimen was implemented. Tumor tissue was extracted and processed to isolate a single-cell suspension. These suspensions were then subjected to staining using fluorescence-conjugated anti-mouse CD11b (eBioscience, APC), F4/80 (eBioscience, PE-cyanine 7) antibodies. Subsequent analysis was performed using a Beckman Coulter flow cytometer.

### In vivo antitumor evaluation

The HCT116 tumor model was established in male BALB/c nude mice. Once the tumor volumes reached approximately 100 mm^3^, the tumor-bearing mice were randomly divided into 12 groups (I: saline, II: CT NPs (12.0 mg/kg), III: MPB NPs + laser (15.0 mg/kg), IV: 3BP (3.0 mg/kg), V: CM NPs (12.0 mg/kg), VI: MPB-3BP NPs + laser (18.0 mg/kg), VII: MPB@CM NPs + laser (27.0 mg/kg), VIII: 3BP@CM NPs (15.0 mg/kg), IX: MPB NPs/3BP/CM NPs + laser (5:1:4, m/m/m, 30.0 mg/kg), X: MPB-3BP@CT NPs + laser (30.0 mg/kg), XI: MPB-3BP@CM NPs (30.0 mg/kg), and XII: MPB-3BP@CM NPs + laser (30.0 mg/kg)). Subsequently, each mouse from the different experimental groups was i.v. injected with 200 μL of the specified drug, with administration taking place every other day for a cumulative total of three doses. In the laser treatment groups, tumors received exposure to an 808 nm laser operating at a power density of 1.0 W/cm^2^ for 5 min following intravenous injection 6 h prior. The body weights and tumor volumes of the nude mice were regularly monitored and recorded. On the 21st d after the initial injection, mice were euthanized and tumors as well as major organs were collected for subsequent H&E, Ki67, and TUNEL staining. Survival analysis spanned a period of 60 days, during which the survival time of each mouse was meticulously recorded. Tumor dimensions were measured using a digital caliper, capturing both length and width, with corresponding volumes calculated via the formula: Volume = Width^2^ × Length/2. Furthermore, the anti-CRC efficacy of MPB-3BP@CM NPs was further evaluated in an HT29 subcutaneous tumor model using the identical treatment protocol.

### Statistics

The animal studies were conducted following randomization. Statistical analyses were performed using GraphPad Prism software (version 8.0). The data were presented as mean ± standard deviation (SD) derived from a minimum of three independent experiments. Differences among groups were assessed using analysis of variance (ANOVA), with statistical significance defined as *P* < 0.05 (*P < 0.05, **P < 0.01, ***P < 0.005, and ****P < 0.0001).

### Supplementary information

Supplementary information

## Data Availability

All data generated or analyzed during this study have been included either in this article or in the supplementary information files. The RNA-seq data has been submitted to the NCBI Sequence Read Archive (SRA) under accession number (PRJNA1105513).
