# Supplementary material for: Genetically programmable cell membrane-camouflaged nanoparticles for targeted combination therapy of colorectal cancer
Source: Signal Transduct Target Ther. 2024 Jun 12;9:158. doi: 10.1038/s41392-024-01859-4 (PMC11167040; doi:10.1038/s41392-024-01859-4)
Supplement: Supplementary file 1 — Supplementary information [file 41392_2024_1859_MOESM1_ESM.docx]

**Supplementary Materials for**

**Genetically Programmable Cell Membrane-Camouflaged Nanoparticles for Targeted Combination Therapy of Colorectal Cancer**

Yun Yang, Qingya Liu, Meng Wang, Lang Li, Yan Yu, Meng Pan, Danrong Hu, Bingyang Chu, Ying Qu, Zhiyong Qian

Correspondence to: Zhiyong Qian (zhiyongqian@scu.edu.cn)

**This PDF file includes:**

Figures. S1 to S21

Tables S1 to S2

**
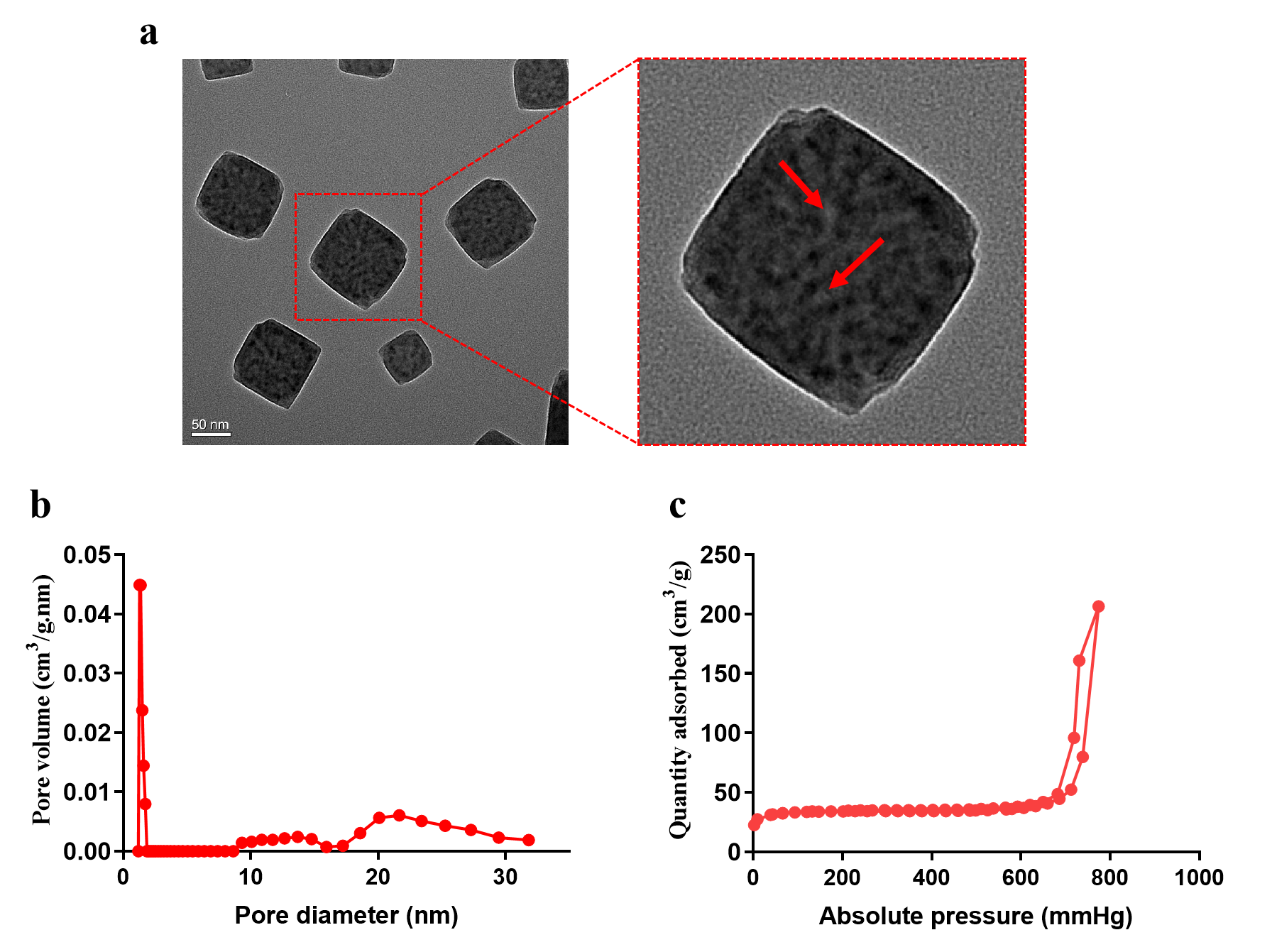
**

**Figure. S1.** (**a**) The TEM image displays the morphology of MPB NPs. The right image is the enlarged one in the red collar on the left images, and the white area pointed by the red arrow corresponds to pores on the surface of the MPB NPS. Scale bar = 50 nm. Pore size distribution (**b**) and N_2_ adsorption/desorption isotherms (**c**) for MPB NP_S_.

**
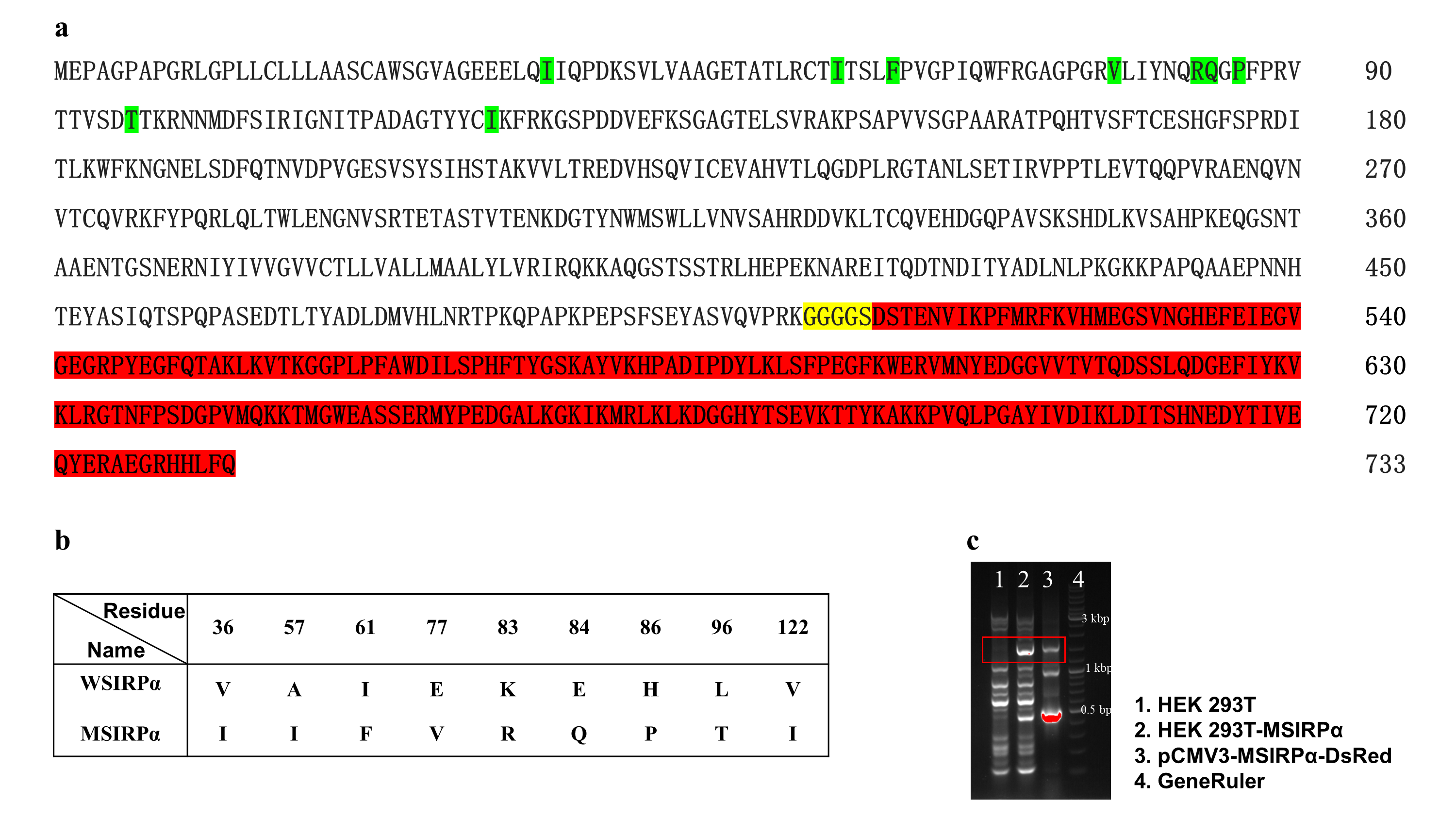
**

**Figure. S2.** (**a**) The amino acid residue sequence of DsRed-MSIRPα. The mutated amino acids are highlighted in green, the linker sequence is highlighted in yellow, and the DsRed protein is highlighted in red. (**b**) The position of the mutated residues and their corresponding sequence in wild-type SIRPα (WSIRPα) and MSIRPα are denoted in table. (**c**) The PCR result of DsRed-MSIRPα gene sequence in the genome of HEK 293T-MSIRPα cell. The pCMV3-MSIRPα-DsRed vector was used as a positive control, and the genome of HEK 293T cell was used as a negative control. The band within the red box represents the DsRed-MSIRPα gene sequence.

**
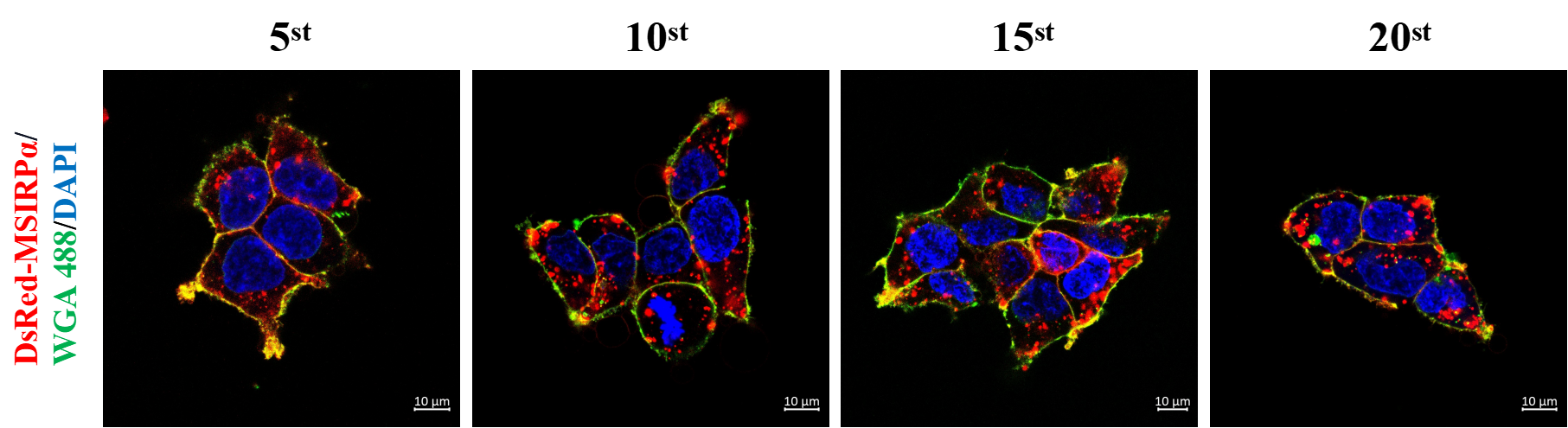
**

**Figure. S3.** The confocal images indicate the expression of DsRed-MSIRPα receptors on the membrane of HEK 293T-MSIRPα cells across various generations. Scale bar = 10 µm.


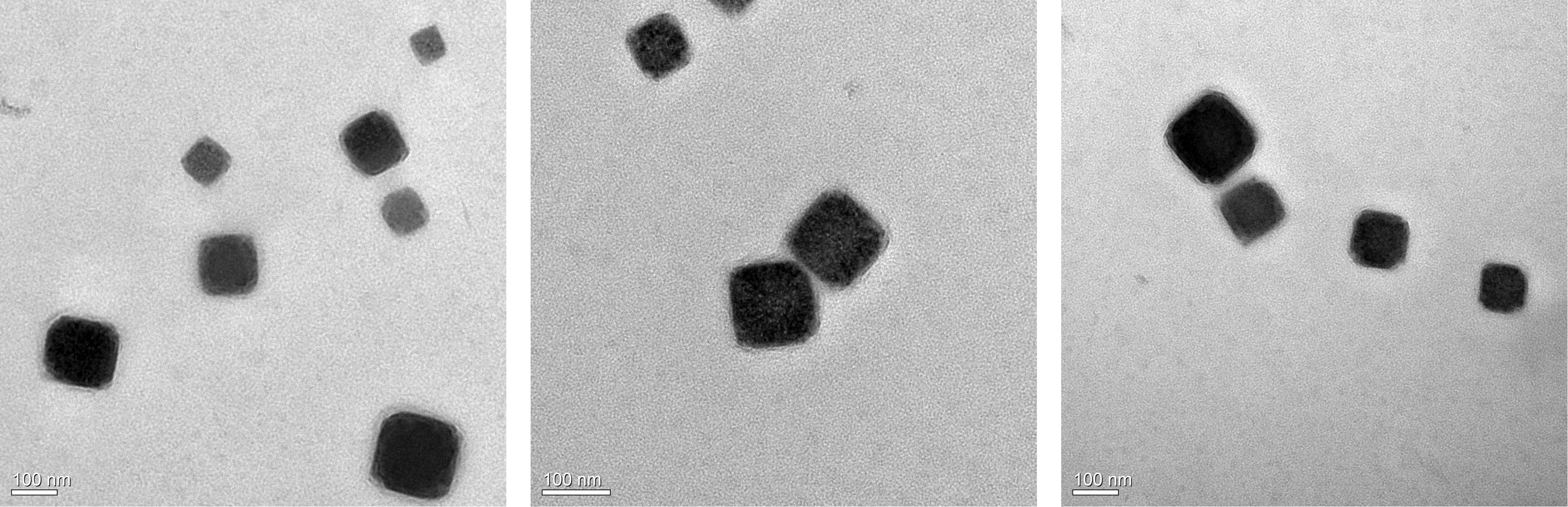


**Figure. S4.** The TEM images of MPB-3BP@CM NPs, scale bar = 100 nm.

**
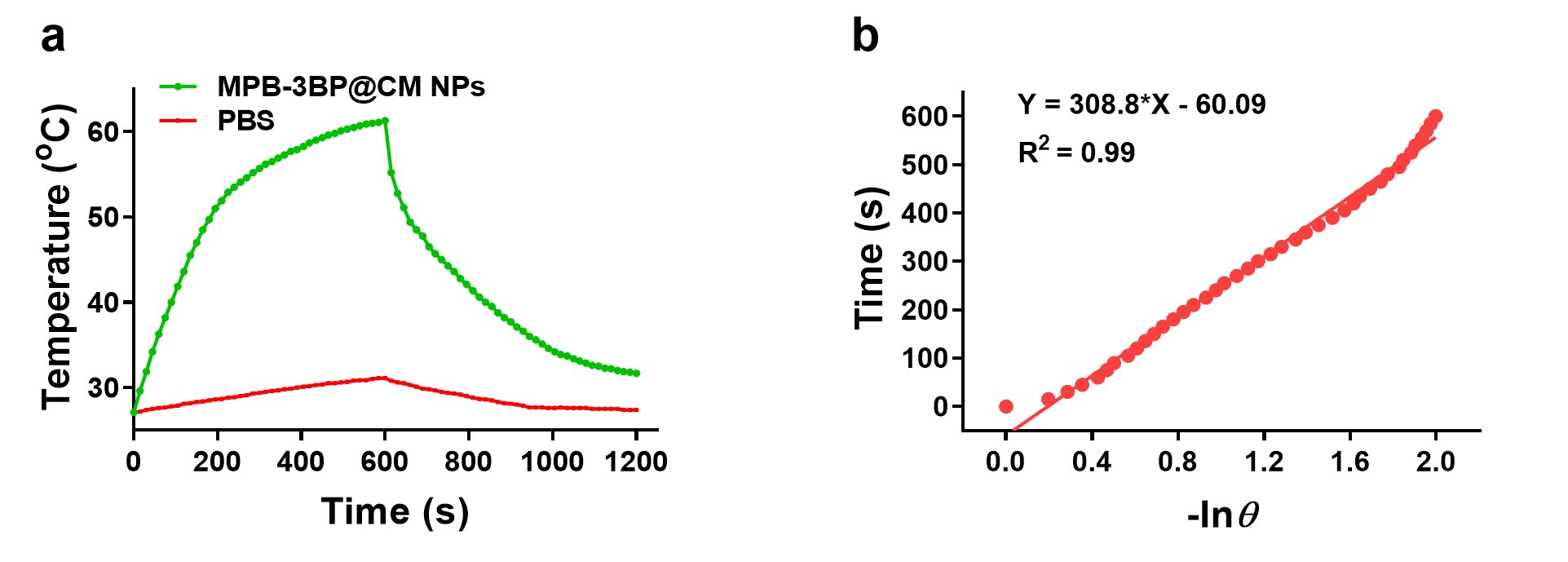
**

**Figure. S5.** Photothermal conversion efficiency of MPB-3BP@CM NPs. (**a**) The temperature curves of MPB-3BP@CM NPs solution under the 808 nm laser irradiation at the power density of 1.0 W/cm^2^ over time. (**b**) Plot of cooling time versus negative natural logarithm of driving force temperature.


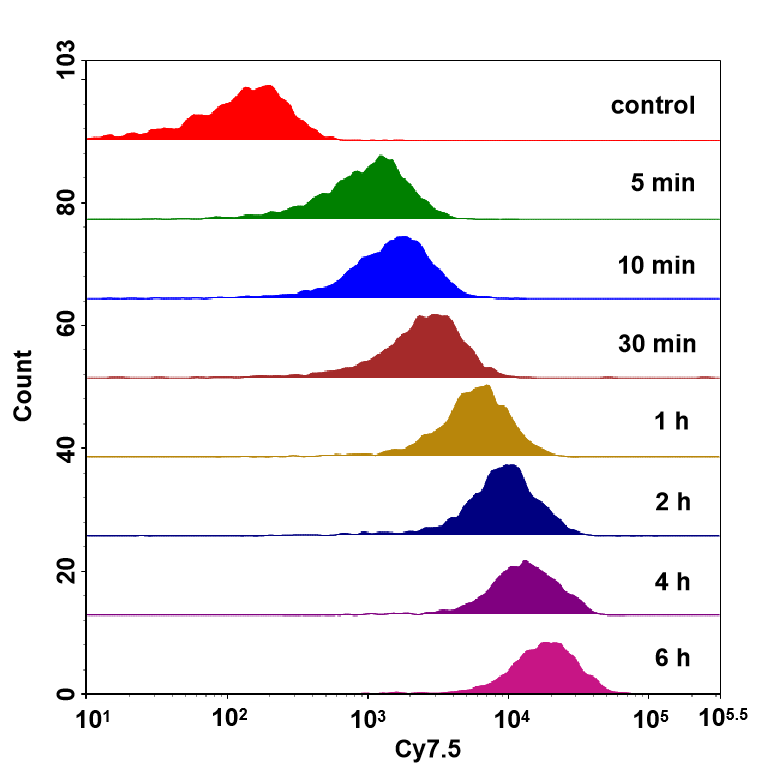


**Figure. S6.** Flow cytometric analysis was conducted on HCT116 cells incubated with MPB-Cy7.5@CM NPs at different time intervals.


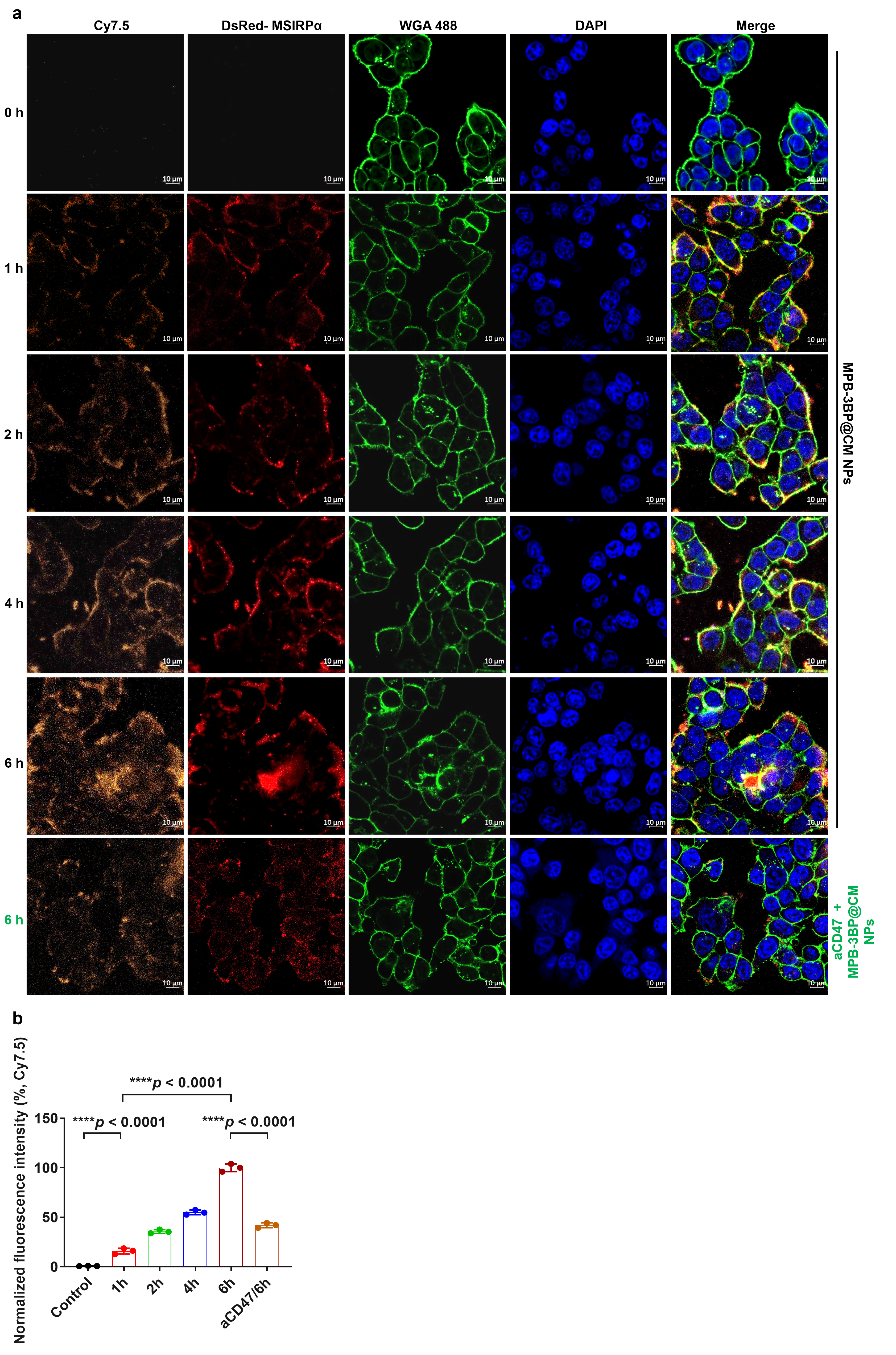
**Figure. S7.** (**a**) CLSM images of HCT116 cells after incubation with MPB-Cy7.5@CM NPs with varying time intervals. The yellow channel fluorescence emission originated from Cy7.5 moieties, the red channel fluorescence emission originated from DsRed moieties. WGA 488 dye was used to detect HCT116 cell membrane (green channel), DAPI was used to detect cell nucleus (blue channel). Scale bar = 10 µm. (**b**) The intracellular normalized fluorescence intensity (%, Cy7.5 channel) of HCT116 cells was quantified from CLSM observations. All data are presented as mean ± S.D. (*n* = 3).


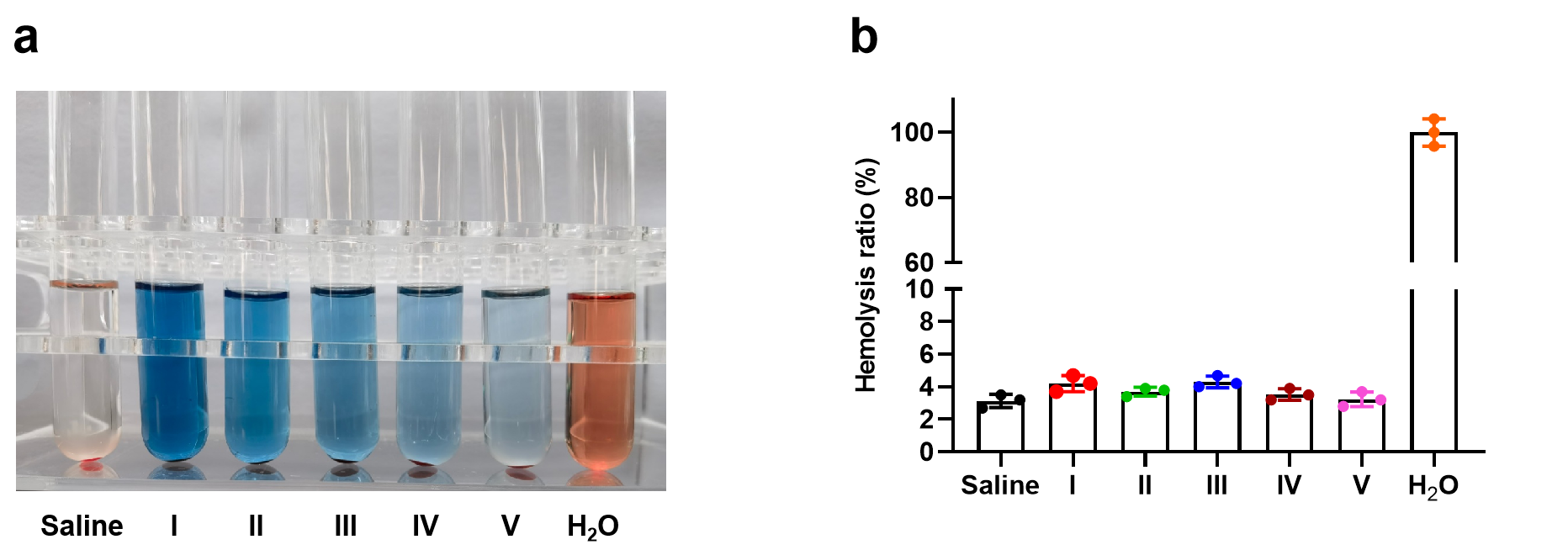


**Figure. S8.** The hemolysis test (**a**) and hemolysis rate (**b**) at different concentrations of MPB-3BP@CM NPs (I: 500.0 μg/mL, II: 200.0 μg/mL, III: 100.0 μg/mL, IV: 50.0 μg/mL, V: 20.0 μg/mL). All data are presented as mean ± S.D. (*n* = 3).


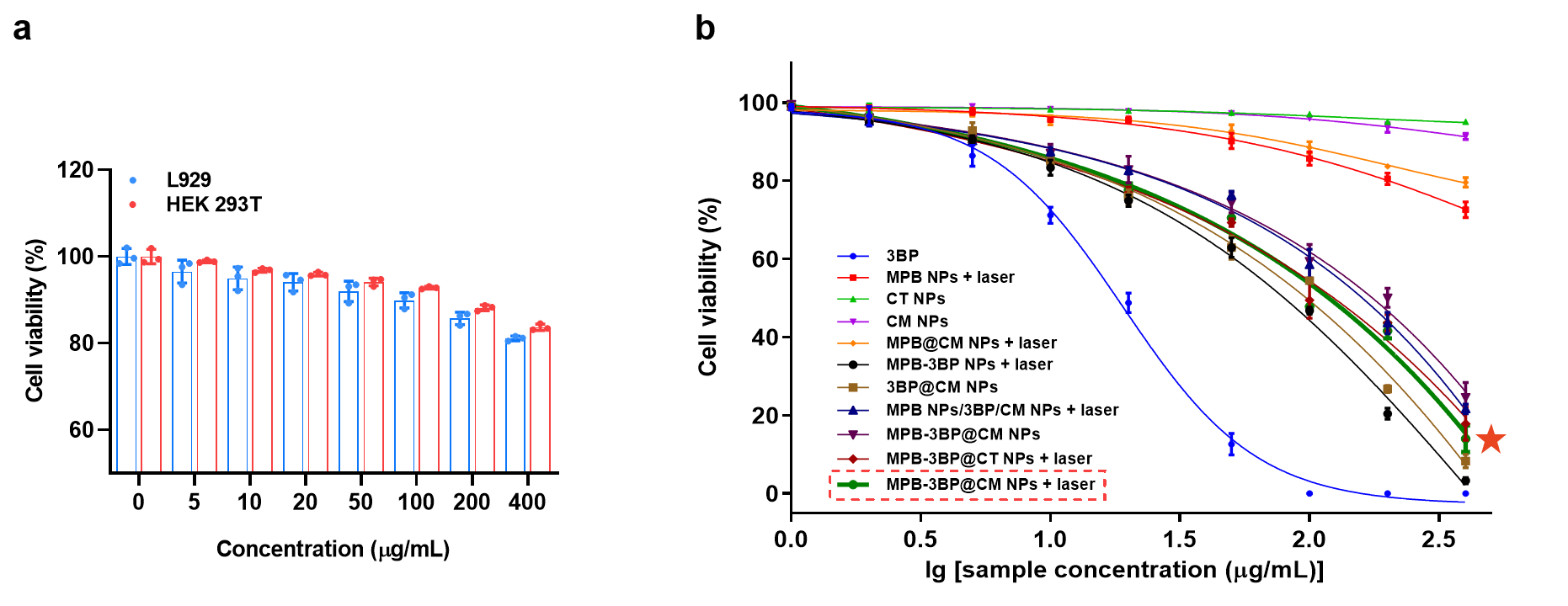


**Figure. S9.** (**a)** The *in vitro* cytotoxicity of MPB-3BP@CM NPs was determined by MTT assay against HEK 293T and L929 cells at varying concentrations. (**b**) The viability of HCT116 cells was assessed *in vitro* using the MTT assay under different treatments. All data are presented as mean ± S.D. (*n* = 3).


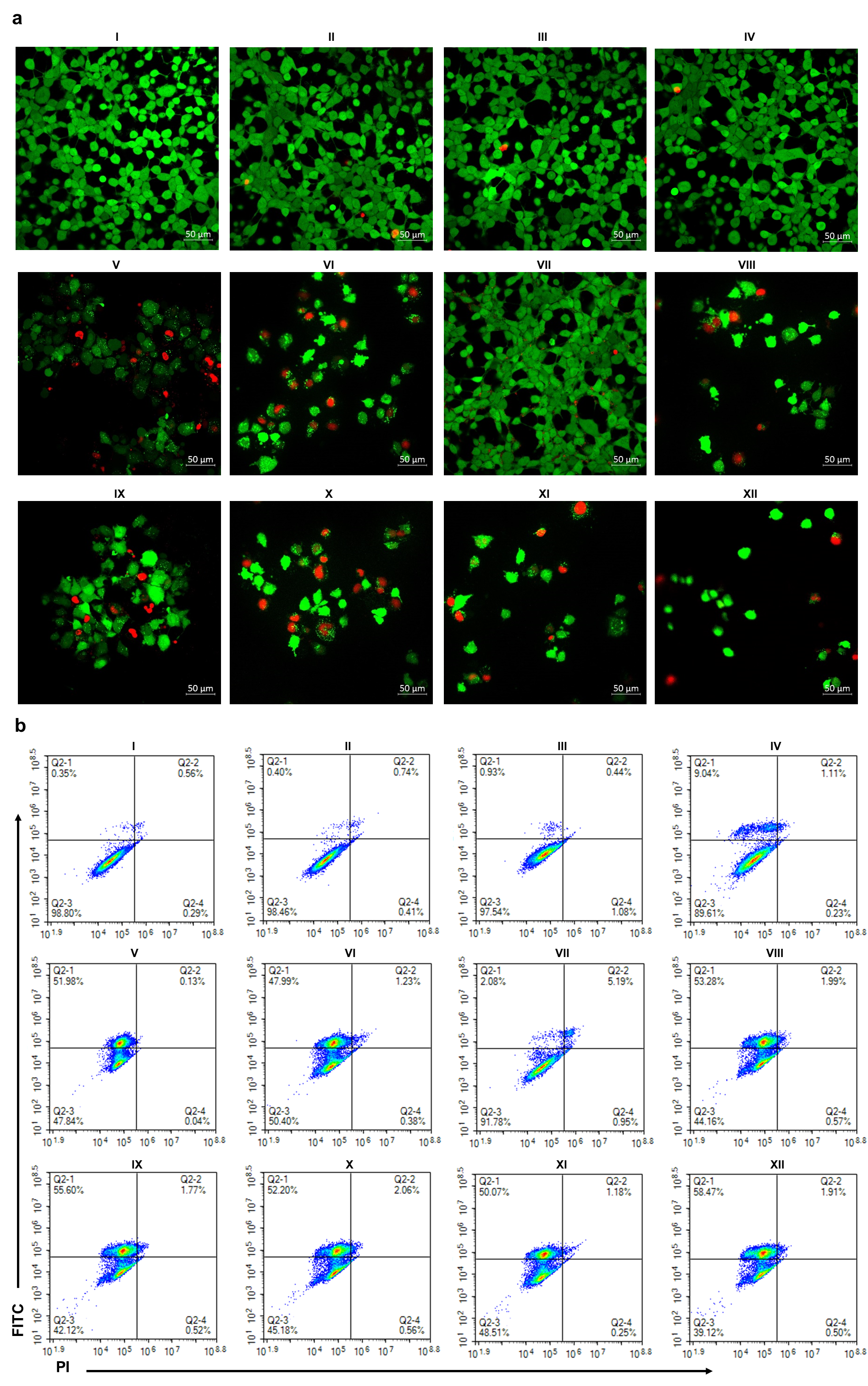
**Figure. S10.** (**a**) Representative CLSM images of Calcein-AM/PI costaining of HCT116 cells after with different treatments (i: PBS, ii: CT NP_s_, iii: CM NP_s_, iv: MPB NP_s_ + laser, v: 3BP, vi: 3BP@CM NPs, vii: MPB@CM NPs + laser, viii: MPB-3BP NP_s_ + laser, IX: MPB NPs/3BP/CM NPs + laser, X: MPB-3BP@CT NPs + laser, XI: MPB-3BP@CM NPs, XII: MPB-3BP@CM NPs + laser). Scale bar = 50 µm. (**b**) Representative flow cytometry plots of apoptosis in HCT116 cells following various treatments.

**
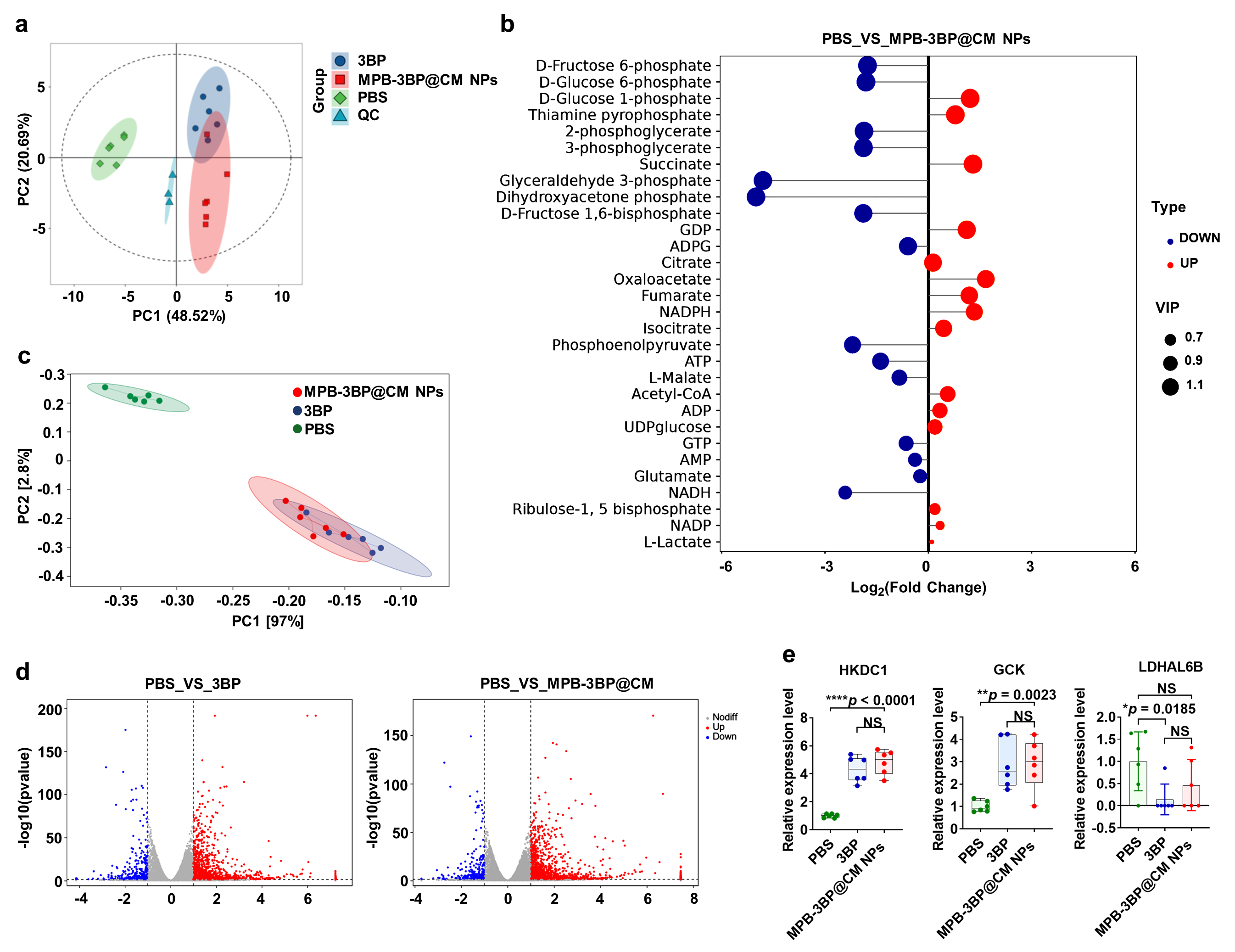
**

**Figure. S11. (a)** Principal Component Analysis (PCA) revealed clustering based on different treatments, with each LC-MS data for each treatment group was color-coded. **(b)** Bubble map comparing the alterations in metabolite content between the control group and the MPB-3BP@CM NPs group. **(c)** PCA revealed clustering based on different treatments, with each RNA-seq data for each treatment group was color-coded. **(d)** Volcano plots were employed to compare the differentially expressed genes between the control and experimental groups. (**e)** The relative expression levels of differentially expressed genes associated with glycolytic pathways were assessed in different groups based on the results of RNA-seq analysis. All data are presented as mean ± S.D. (n = 6).


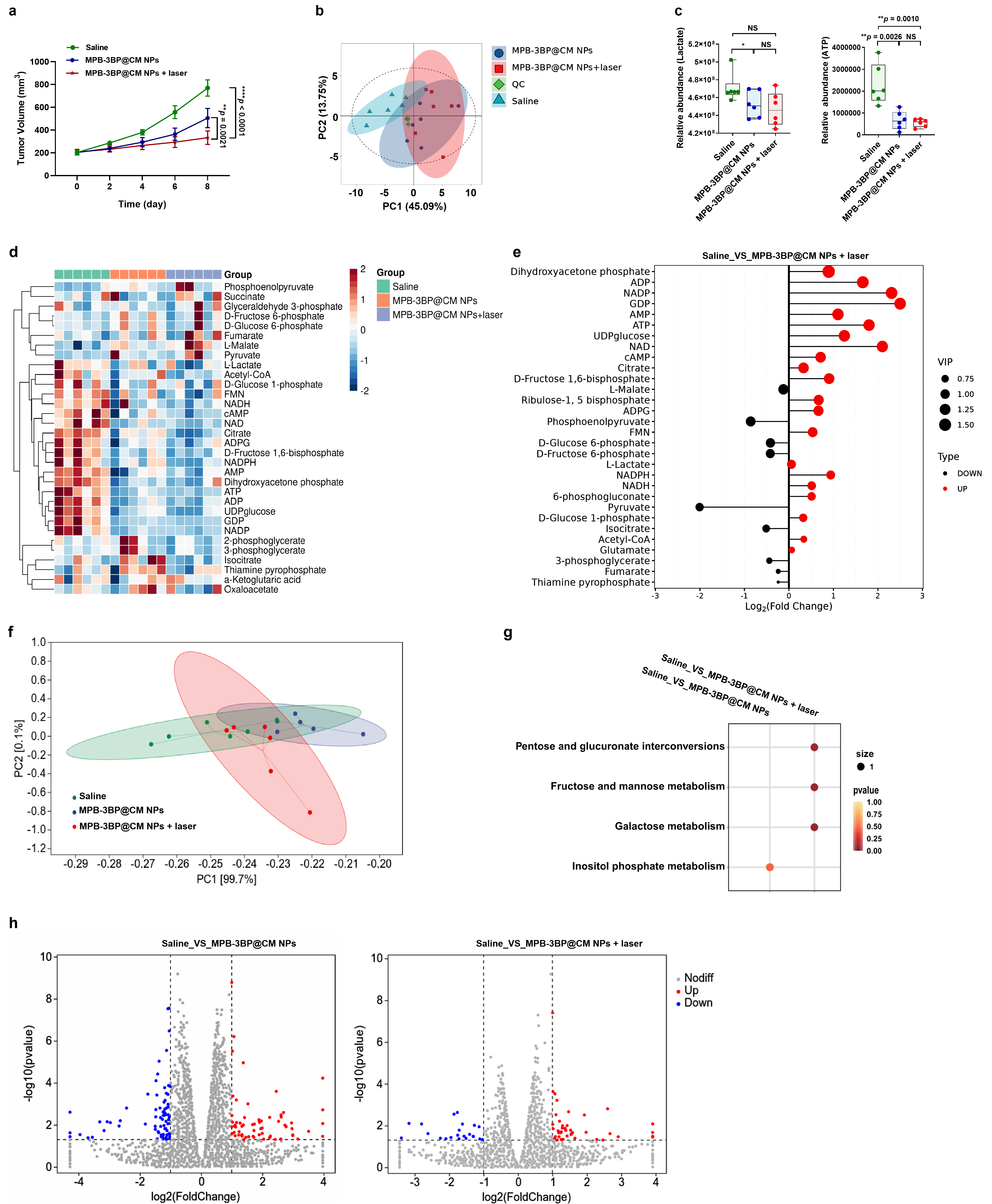


**Figure. S12.** (**a**) The growth curves of tumors in different groups. (**b**) PCA revealed clustering based on different treatments, with each LC-MS data for each treatment group was color-coded. (**c**) The relative levels of ATP and lactate in different groups were quantified by LC-MS. (**d**) Heat map illustrates the heterogeneity in metabolite levels across different groups. (**e**) Bubble map comparing the alterations in metabolite content between the saline group and the MPB-3BP@CM NPs + laser group. (**f**) PCA revealed clustering based on different treatments, with each RNA-seq data for each treatment group was color-coded. (**g**) Bubble map were employed to compare the differentially expressed genes associated with metabolism between the control and experimental groups. (**h**) Volcano plots were employed to compare the differentially expressed genes between the control and experimental groups. All data are presented as mean ± S.D. (n = 6).


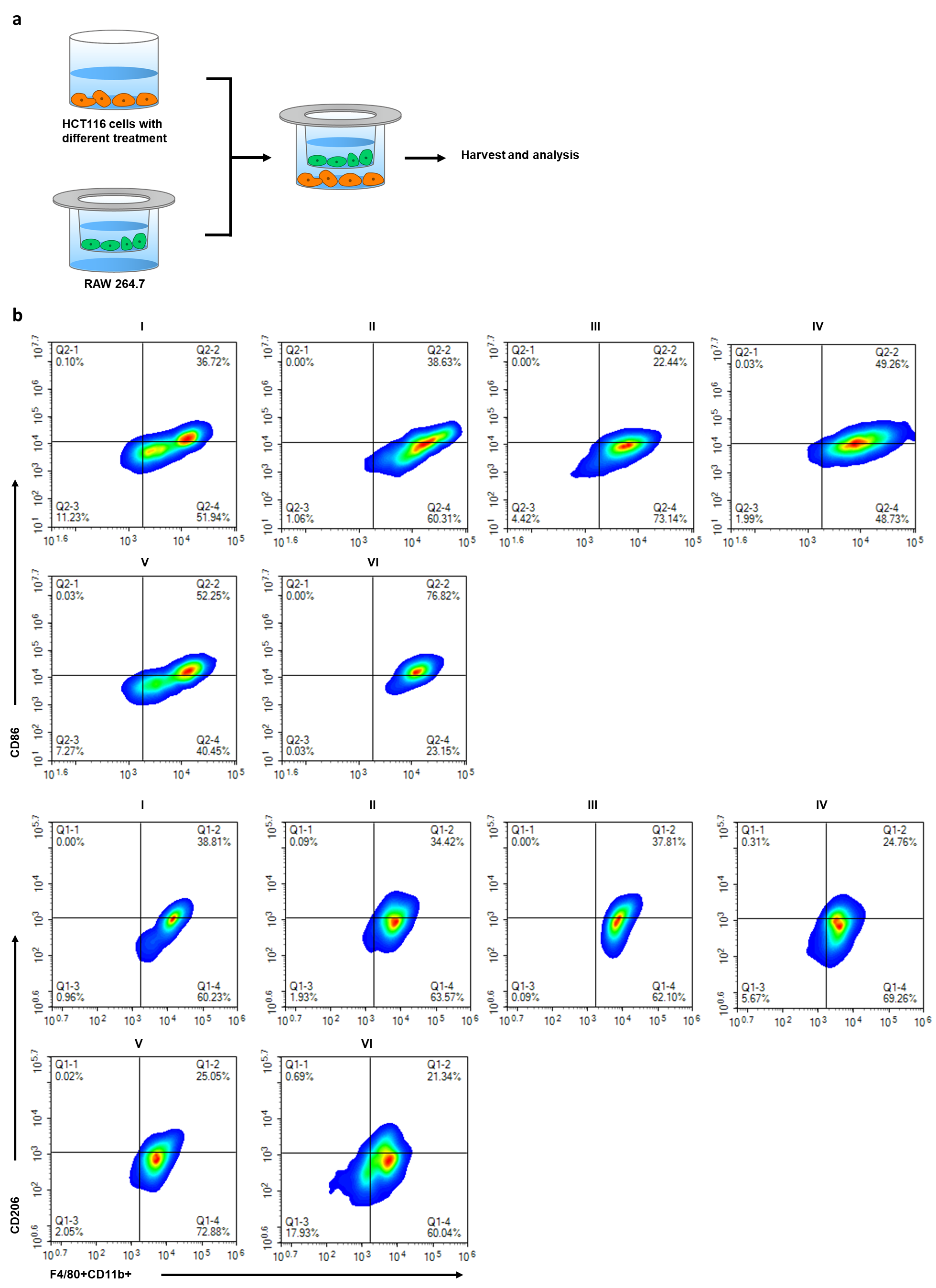


**Figure. S13. (a)** Schema for the representation of experiment procedures. **(b)** Representative flow cytometry plots of M1-like macrophages (CD86+) and M2-like macrophages (CD206+) in RAW264.7 gating on F4/80+CD11b+ cells (i: PBS, ii: Cm NP_s_, iii: MPB NP_s_ + laser, iv: 3BP, v: MPB-3BP@CM NPs, vi: MPB-3BP@CM NPs + laser).


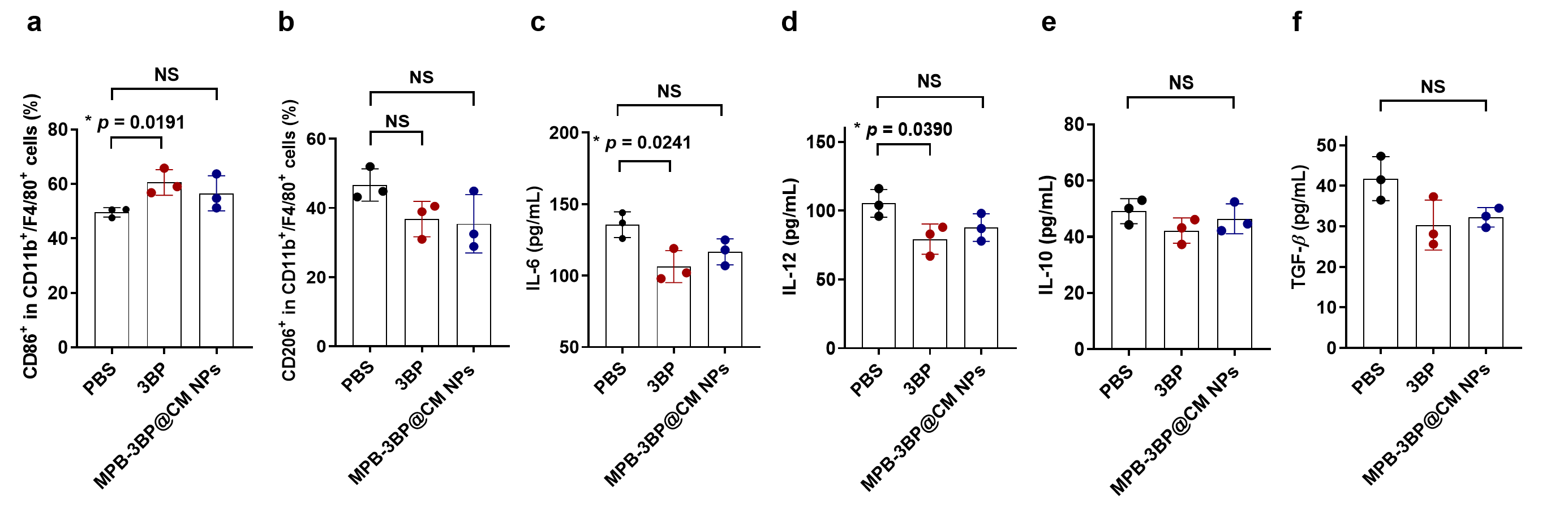
**Figure. S14.** The flow cytometric analysis was performed to quantify both M1-like macrophages (CD86+) (**a)** and M2-like macrophages (CD206+) (**b)** were quantified in RAW264.7 gating on F4/80+CD11b+ cells. ELISA measurement of the secretion of IL-6 (**c)**, IL-12 (**d)**, IL-10 (**e)** and TGF-β (**f)** in different treatment groups. All data are presented as mean ± S.D. (n = 3).


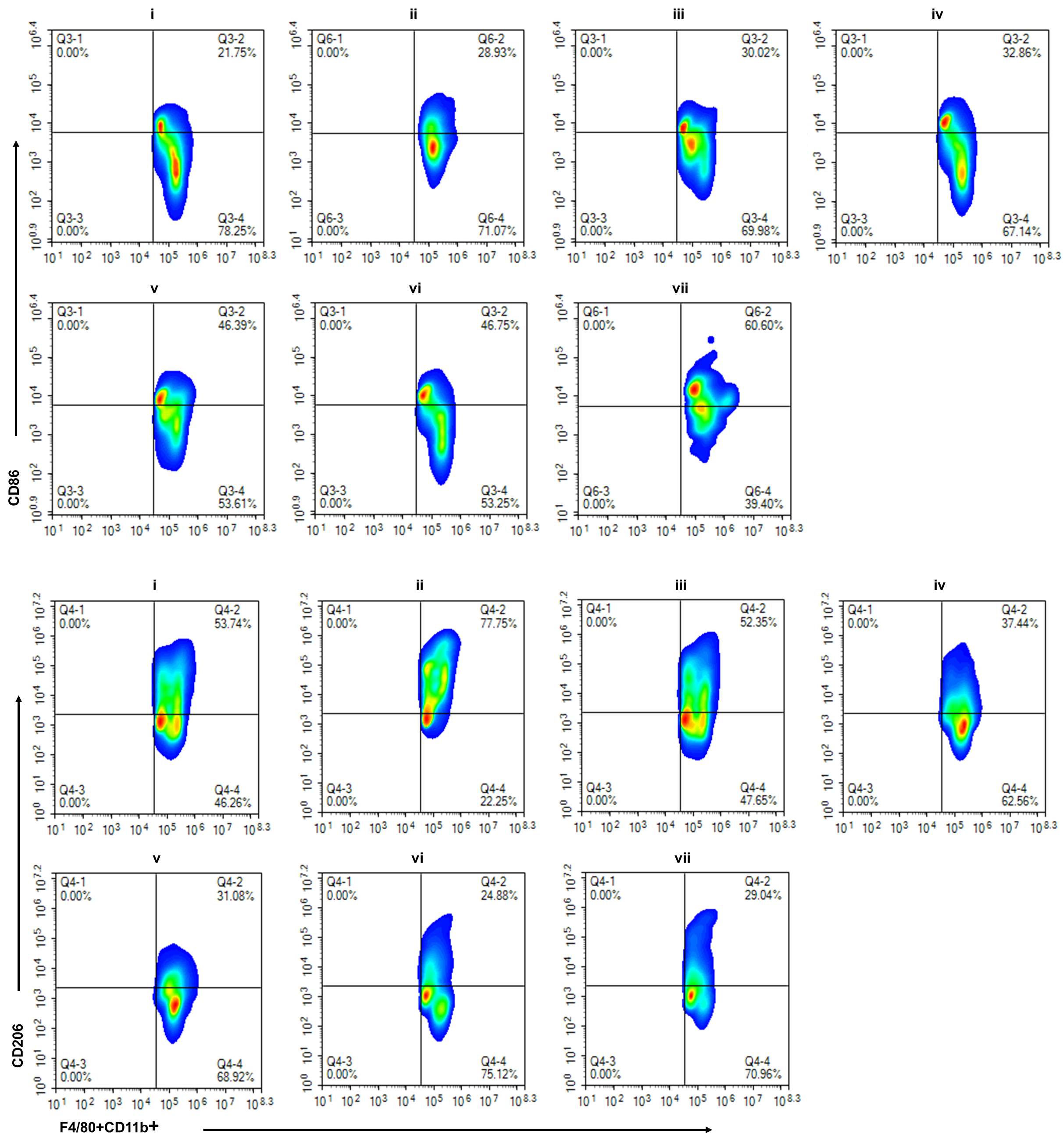
**Figure. S15.** Representative flow cytometry plots of M1-like macrophages (CD86+) and M2-like macrophages (CD206+) in tumor gating on F4/80+CD11b+ cells (i: saline, ii: MPB NPs + laser, iii: 3BP, iv: CM NPs, v: MPB-3BP@CT NPs + laser, vi: MPB-3BP@CM NPs, vii: MPB-3BP@CM NPs + laser).


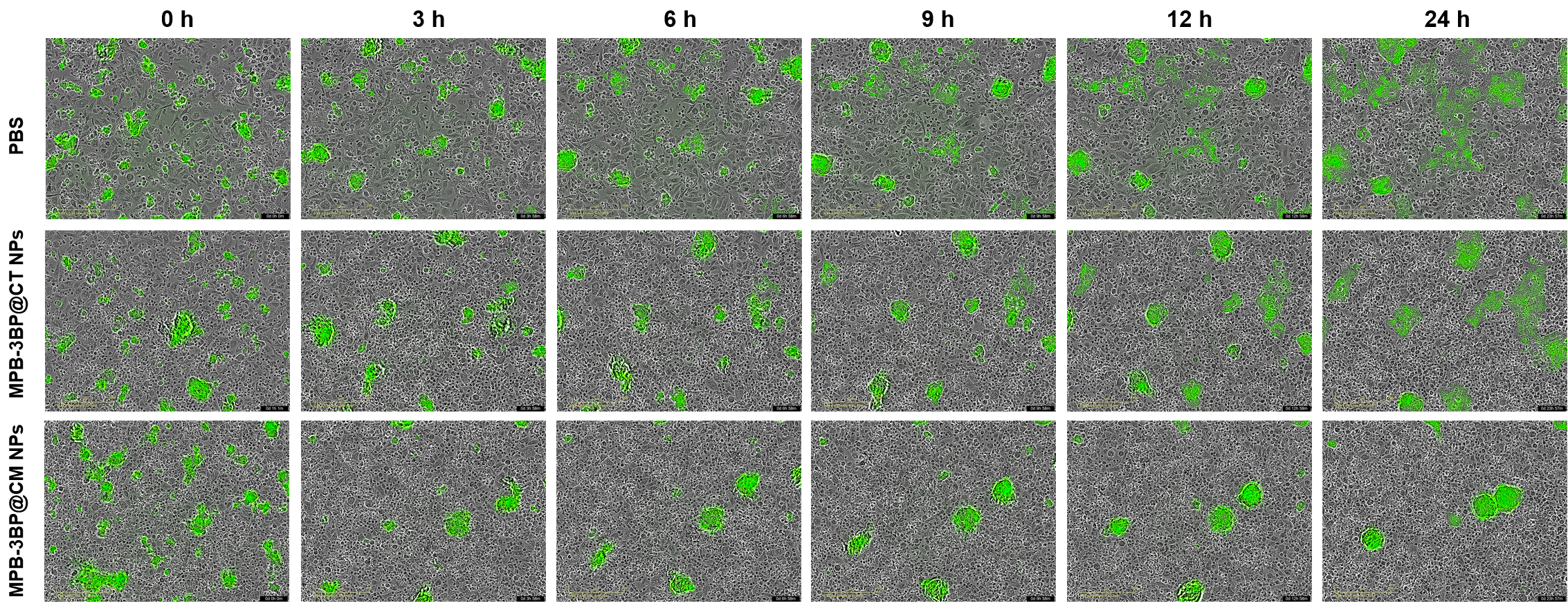
**Figure. S16.** Representative confocal images of the phagocytosis of HCT116-EGPF cells by BMDMs after different treatments *in vitro*. The confocal images within each group were captured at a consistent location across multiple time points. Scale bar = 200 µm.


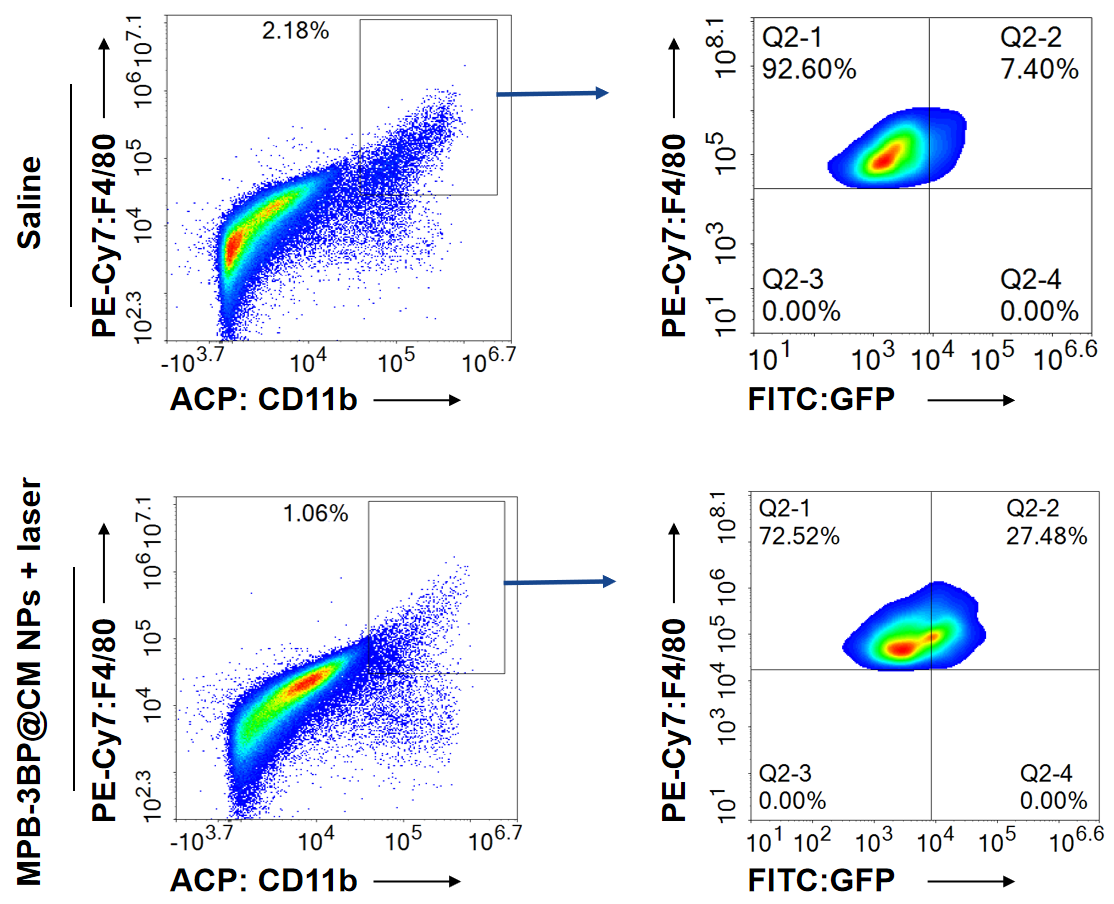


**Figure. S17.** Gating strategy for *in vivo* phagocytosis assay. The phagocytosis efficiency is represented by the percentage of CFSE+F4/80+CD11b+ cells in total F4/80+CD11b+ cells.


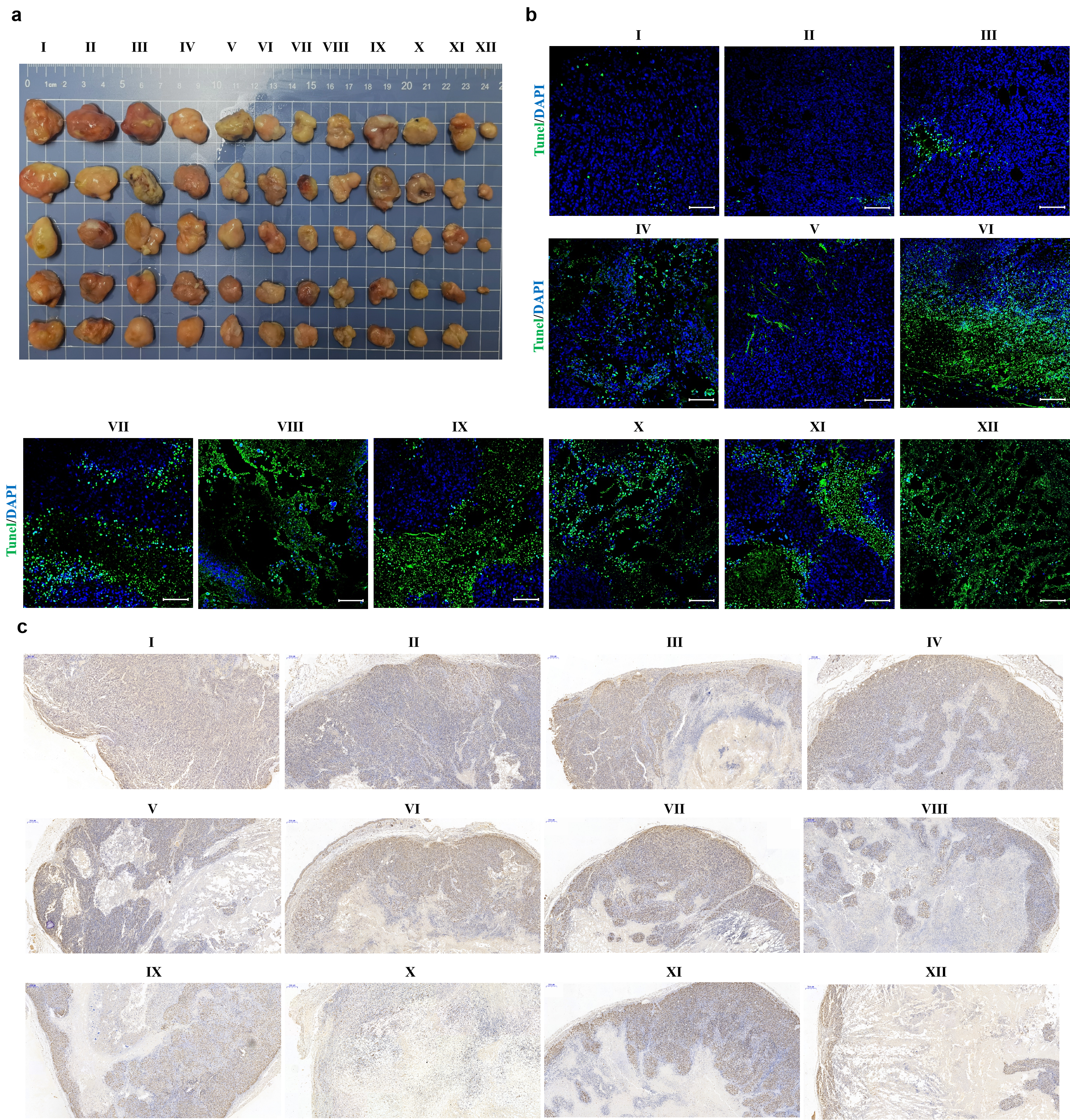
**Figure. S18.** (**a**) Photographs of subcutaneous tumors in each group (I: saline, II: CT NPs, III: MPB NPs + laser, IV: 3BP; V: CM NPs, VI: MPB-3BP NPs + laser, VII: MPB@CM NPs + laser, VIII: 3BP@CM NPs, IX: MPB NPs/3BP/CM NPs + laser, X: MPB-3BP@CT NPs + laser, XI: MPB-3BP@CM NPs, XII: MPB-3BP@CM NPs + laser). (**b**) Representative TUNEL staining of tumor in each group, scale bar = 50 µm. (**c**) Representative Ki-67 staining of tumor in each group, scale bar = 200 μm.


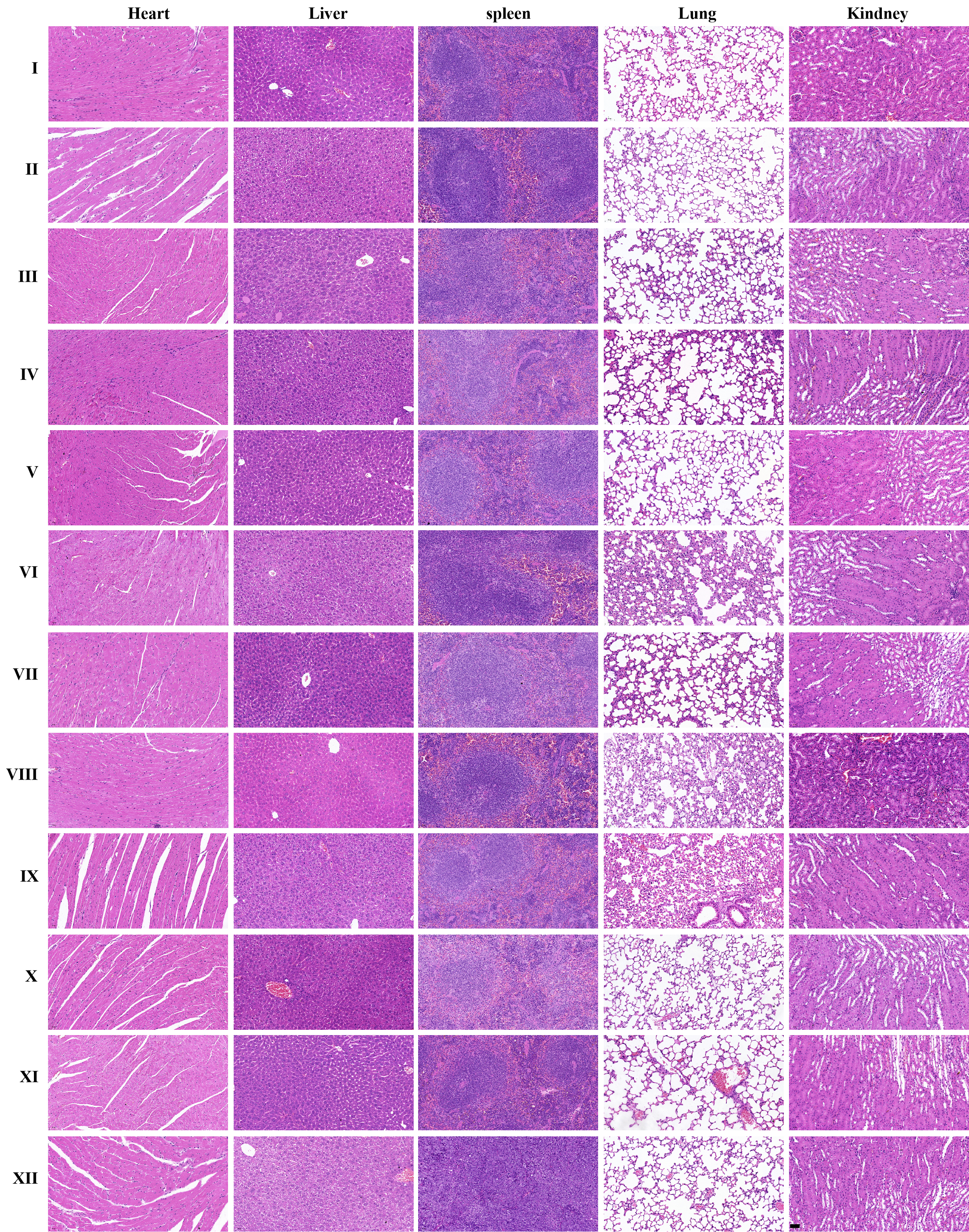
**Figure. S19.** Representative H&E stained images of the liver, heart, spleen, lung, and kidney tissues after different treatments (I: saline, II: CT NPs, III: MPB NPs + laser, IV: 3BP, V: CM NPs, VI: MPB-3BP NPs + laser, VII: MPB@CM NPs + laser, VIII: 3BP@CM NPs, IX: MPB NPs/3BP/CM NPs + laser, X: MPB-3BP@CT NPs + laser, XI: MPB-3BP@CM NPs, XII: MPB-3BP@CM NPs + laser). Scale bar = 50 μm.


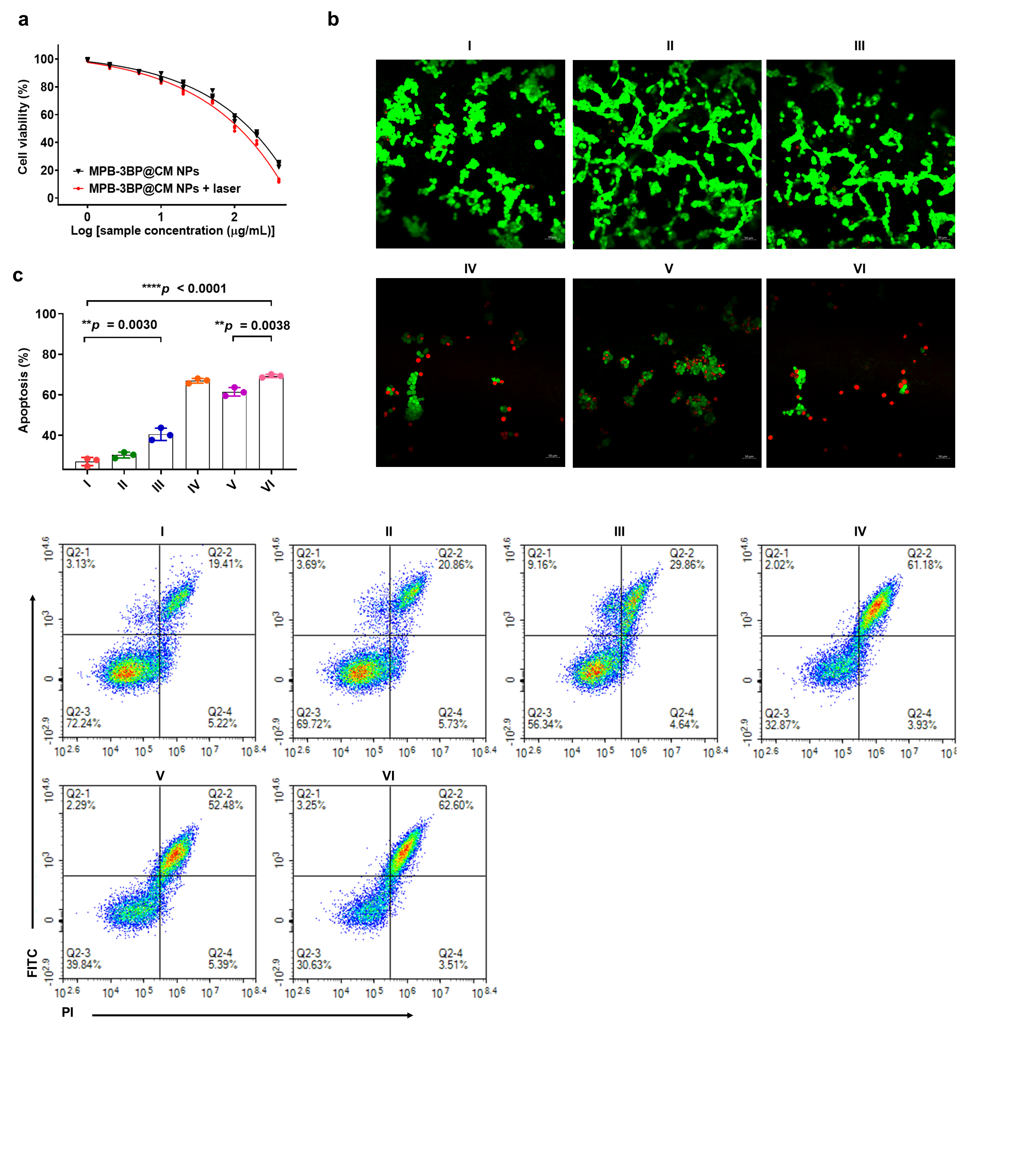


**Figure. S20.** (**a)** The viability of HT29 cells was assessed in vitro using the MTT assay under different treatments. (**b)** CLSM images of Calcein-AM/PI costaining of HT29 cells after with different treatments (i: PBS, ii: CM NP_s_ (80.0 μg/mL), iii: MPB NP_s_ + laser (100.0 μg/mL), iv: 3BP (20.0 μg/mL), v: MPB-3BP@CM NPs (200.0 μg/mL), vi: MPB-3BP@CM NPs + laser (200.0 μg/mL)). Scale bar = 50 µm. (**c)** Representative flow cytometry and quantitative flow cytometry analysis of apoptosis in HT29 cells following various treatments. All data are presented as mean ± S.D. (n = 3).


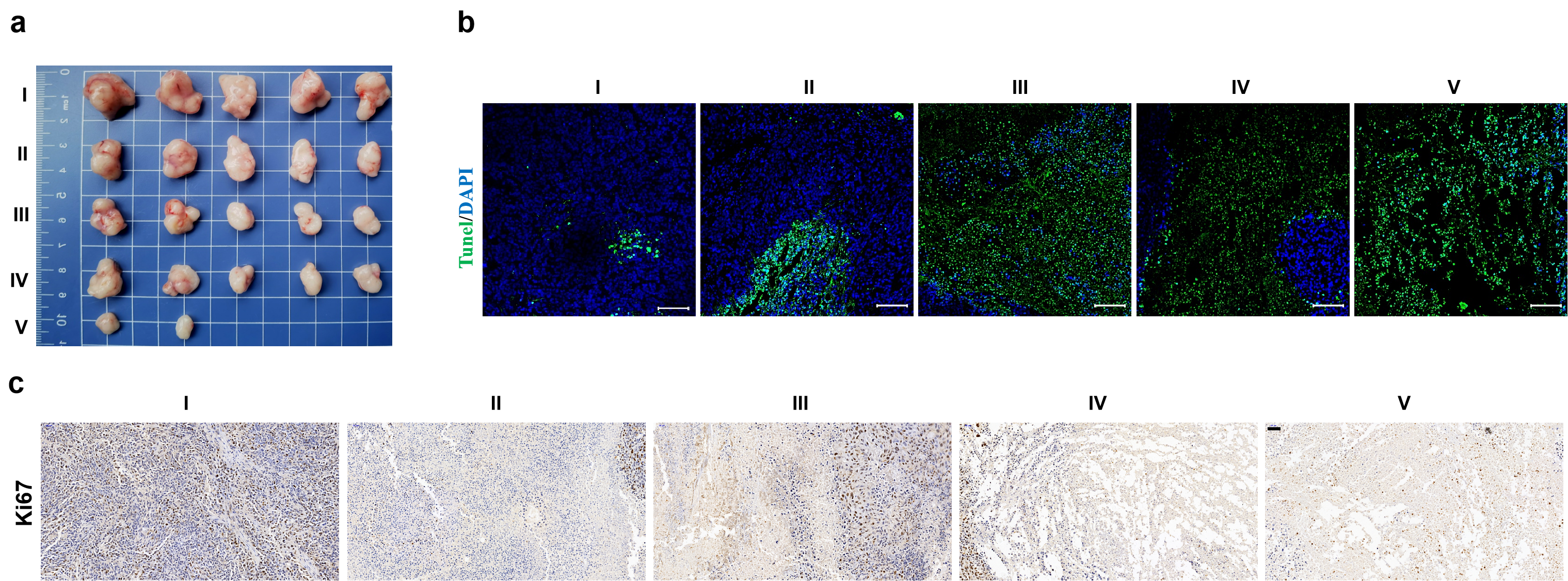
**Figure. S21.** (**a**) Photographs of subcutaneous tumors in each group (I: saline, II: MPB NPs/3BP/CM NPs + laser, III: MPB-3BP@CT NPs + laser, IV: MPB-3BP@CM NPs, and V: MPB-3BP@CM NPs + laser). Representative TUNEL staining (**b**) and Ki-67 staining (**c**) of tumor in each group, scale bar = 50 µm.

**Table S1.** The feeding ratio of MPB NPs and 3BP.

| MPB NPs  (mg) | 3BP dissolved in 4 mL deionized water  (mg/mL) | 3BP content  (%) *^a^* |
| --- | --- | --- |
| 2 | 2 | 2.1 ± 0.8 |
| 2 | 5 | 4.8 ± 1.1 |
| 2 | 10 | 9.5 ± 3.2 |
| **2** | **20** | **16.8 ± 4.6** |
| 2 | 25 | 17.4 ± 5.1 |
| 2 | 30 | 18.8 ± 3.8 |

*^a^* The content of 3BP of MPB-3BP NPs was determined by HPLC. All data are presented as mean ± S.D. (n = 3).

**Table S2.** *In vivo* toxicology data.

|  | Reference range *^a^* | Saline | MPB-3BP@CM NPs (60.0 mg/kg) | |
| --- | --- | --- | --- | --- |
|  |  |  | 1 day | 7 days |
| WBC (10^9^/L) | 0.8 – 6.8 | 2.0 ± 0.8 | 3.2 ± 1.2 | 3.7 ± 0.6 |
| RBC (10^12^/L) | 6.36 – 9.42 | 9.36 ± 0.4 | 9.2 ± 0.6 | 9.41 ± 0.5 |
| HGB (g/L) | 110 – 143 | 143 ± 1.2 | 141.7 ± 2.4 | 135 ± 4.5 |
| PLT (10^9^/L) | 450 – 1590 | 588 ± 15.0 | 476.7 ± 25.4 | 903 ± 91.5 |

*^a^* Reference ranges of hematology data of healthy male Balb/c nude mice were obtained from Chengdu Li Lai Biotechnology Co., Ltd. All data are presented as mean ± S.D. (*n* = 5).
